# The Therapeutic Potential of Exosomes in Ocular Surface Diseases

**DOI:** 10.3390/biom16040512

**Published:** 2026-03-30

**Authors:** Lanxin Hu, Hongyu Duan, Yu Zhang, Liang Yang, Kyung Chul Yoon, Zihan Shen, Zekai Li, Baikai Ma, Hong Qi

**Affiliations:** 1 Beijing Key Laboratory of Restoration of Damaged Ocular Nerve, Department of Ophthalmology, Peking University Third Hospital, 49 North Garden Rd., Haidian District, Beijing 100191, China; 2110301317@stu.pku.edu.cn (L.H.); 2311110518@stu.pku.edu.cn (H.D.); zhangyu2020@zju.edu.cn (Y.Z.); 2511110517@stu.pku.edu.cn (L.Y.); 2 Department of Ophthalmology, Chonnam National University Medical School and Hospital, Gwangju 61469, Republic of Korea; kcyoon@jnu.ac.kr; 3 Institute of Medical Technology, Peking University Health Science Center, Beijing 100191, China; shenzihan@bjmu.edu.cn (Z.S.); 2411210768@stu.pku.edu.cn (Z.L.)

**Keywords:** exosomes, extracellular vehicles, ocular surface diseases

## Abstract

The ocular surface system, essential for maintaining visual function, is highly susceptible to a range of ocular surface diseases (OSDs) that significantly impair patients’ quality of life. Current treatments for OSDs often face limitations including low bioavailability, A lack of targeted delivery, and an inadequate capacity to fully address the complex pathophysiology involving inflammation, oxidative stress, and impaired tissue repair. In recent years, exosomes have emerged as promising cell-free therapeutic platforms for OSDs. This review evaluates their therapeutic potential across the OSD spectrum, focusing on three key aspects: mechanisms—modulation of inflammation, oxidative stress, and tissue repair via bioactive cargo; applications—preclinical therapeutic effects in dry eye disease, corneal injury, keratitis, and transplant rejection; and optimization strategies—engineering approaches and biomaterial integration to enhance stability, targeting, and ocular retention. We also discuss critical challenges in standardization, scalable production, and clinical translation, highlighting future directions for exosome-based OSD therapies.

## 1. Introduction

The ocular surface is a highly integrated system composed of the cornea, conjunctiva, tear film, meibomian glands, and associated neural and immune networks [1]. It plays a vital role in maintaining visual function by ensuring optical clarity, hydration, and mechanical protection, while also serving as a physical and immunological barrier against external insults. The integrity of this system is essential for visual health, yet it is susceptible to disruption by various factors such as infection, trauma, autoimmune disorders, surgical interventions, aging, and lifestyle factors like prolonged screen exposure. Such disruptions can lead to ocular surface diseases (OSDs), a group of disorders that impair the structure and function of the ocular surface, including superficial corneal disorders, conjunctival diseases, external ocular conditions, and lacrimal gland/duct disorders [2]. Common OSDs include dry eye disease (DED), corneal injury, keratitis, and conjunctivitis, which are associated with significant discomfort, pain, and visual impairment, substantially reducing patients’ quality of life [3]. The prevalence of OSDs is considerable. For example, DED alone affects 5–50% of the population, with incidence increasing with age, posing a substantial public health challenge [4].

Current clinical management of OSDs primarily relies on artificial tears, anti-inflammatory medications, autologous serum, and surgical interventions. However, substantial limitations are associated with each approach: the preparation of autologous serum is complex and carries potential risks, while surgical treatments are inherently invasive [5,6]. Furthermore, the efficacy of traditional topical ocular drug delivery is compromised by anatomical and physiological barriers, resulting in low bioavailability and necessitating frequent administration. In contrast, systemic oral or intravenous routes lack precise ocular surface targeting [7]. Consequently, the development of novel therapeutic strategies capable of achieving targeted tissue regeneration, modulation of immune-inflammatory responses, and robust safety profiles represents an urgent unmet need.

In recent years, extracellular vesicles (EVs), particularly exosomes, have garnered significant attention in regenerative medicine as key mediators of intercellular communication. Exosomes are defined as actively secreted, nanoscale (30–150 nm) membrane vesicles that carry a rich cargo of bioactive substances, including proteins, lipids, mRNA, miRNA, and DNA fragments [8]. These functional cargoes are delivered from source cells to recipient cells, thereby regulating the biological functions of the latter. Accumulating evidence indicates that exosomes derived from mesenchymal stem cells (MSCs), epithelial cells, and other sources inherit numerous therapeutic properties of their parent cells, such as potent anti-inflammatory, antioxidant, anti-fibrotic, and pro-regenerative capabilities, demonstrating significant therapeutic potential [9]. Compared to therapies utilizing MSCs directly, exosomes exhibit low immunogenicity due to their lack of immunogenic surface markers. Their small size facilitates penetration of biological barriers, enhancing bioavailability. Relative to conventional pharmacotherapies, exosomes offer high stability conferred by their lipid bilayer membrane, alongside natural targeting properties enabled by membrane proteins capable of binding to receptors on target cells [10]. Furthermore, as drug delivery platforms, exosomes offer distinct advantages over synthetic carriers such as liposomes or polymeric nanoparticles. Owing to their endogenous origin, they exhibit superior biocompatibility, lower immunogenicity, and intrinsic immuno-modulatory effects [11].

Given this exceptional profile, exosomes represent a highly promising platform for cell-free therapy. Studies on exosomes have expanded rapidly, with applications emerging in oncology, cardiology, neurology, and other fields. Within OSD treatment, the use of exosomes as therapeutic agents or drug delivery vehicles has garnered increasing attention, as evidenced by several published reviews. For instance, Tian et al. [12] summarized the use of exosomes as delivery vehicles in traumatic and autoimmune diseases as well as chorioretinal disorders. Liu et al. [13] discussed the roles of exosomes in conditions such as diabetic retinopathy, age-related macular degeneration, autoimmune uveitis, glaucoma, traumatic optic neuropathy, corneal diseases, retinopathy of prematurity, and uveal melanoma, with an emphasis on oxidative stress. Khorrami-Nejad et al. [10] critically evaluated the therapeutic advantages and mechanisms of MSC-derived exosomes in anterior segment diseases including DED, Sjögren’s syndrome (SS), and corneal graft rejection. However, a systematic review dedicated to the therapeutic application of exosomes across the spectrum of OSDs remains lacking.

Therefore, this review aims to comprehensively evaluate the potential of exosomes in treating OSDs and to summarize their underlying mechanisms. It begins with an overview of exosome composition, biogenesis, biological characteristics, functions, and isolation methods. Focusing on immune inflammation, oxidative stress, tissue repair, and regeneration, the review synthesizes recent advances in the application of exosomes and other EVs to conditions such as DED, corneal injury, keratoconus, keratitis, allergic conjunctivitis, pterygium, and transplant rejection. Additionally, strategies for integrating exosomes with other components to optimize ocular surface drug delivery systems are thoroughly summarized. Finally, perspectives on current limitations, challenges in clinical translation, and future directions are also presented, with the aim of providing a theoretical foundation and novel insights for developing next-generation exosome-based innovative therapies for OSDs.

## 2. Search Strategy

**Figure 1:**
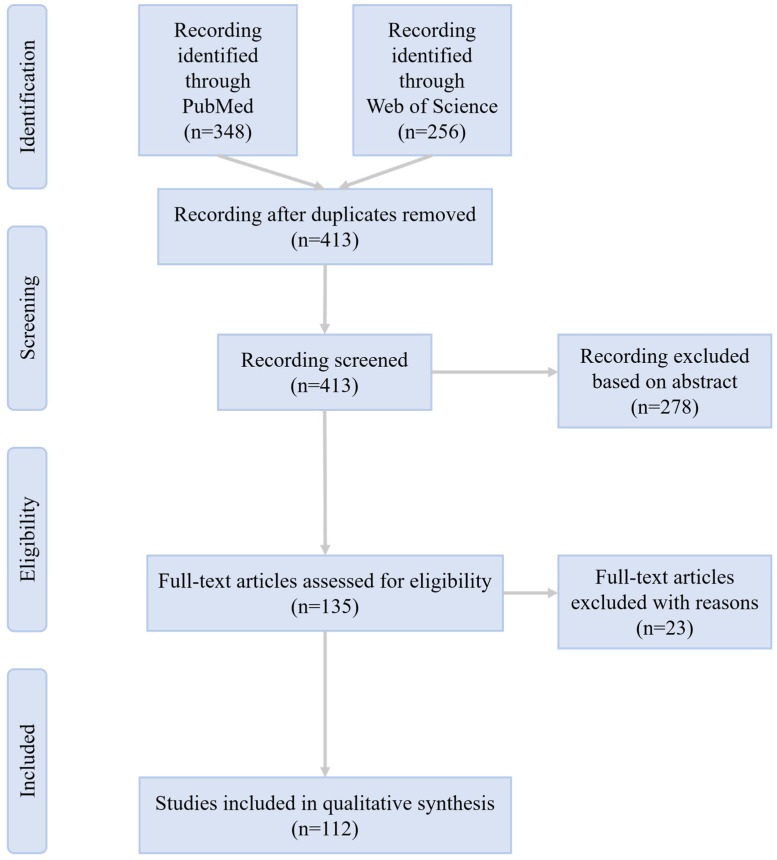
Flow chart of the publication selection process.

We employed a comprehensive literature search strategy to ensure the inclusion of relevant studies on exosomes and their therapeutic potential for OSDs. We utilized a combination of keywords including “exosome”, “extracellular vesicles”, “ocular surface disease”, “dry eye”, “keratoconjunctivitis sicca”, “meibomian gland”, “limbal stem cell”, “cornea”, “cornea injury”, “keratitis”, “corneal dystrophy”, “keratoconus”, “corneal degeneration”, “intraepithelial epithelioma”, “conjunctiva”, “pterygium”, “conjunctivitis”, “subconjunctival hemorrhage”, “lacrimal apparatus”, “dacryoadenitis” and “eyelid” to search articles in PubMed and Web of Science databases. Overall, 112 articles were included, most of which were published in English or translated into English between 2020 and 2025. The study selection process is detailed in the flowchart (Figure 1). Representative studies on exosome-based therapies for ocular surface diseases are summarized in Table 1, including detailed information on exosome sources, isolation methods, and disease models.

## 3. Brief Overview of Exosomes

### 3.1. Exosomes and Extracellular Vesicles

EVs constitute a heterogeneous population of membrane-bound particles that are released by cells and are capable of transporting diverse bioactive molecules, including proteins, lipids, and nucleic acids [8]. These vesicles are ubiquitous in various bodily fluids. Classification into subtypes such as exosomes, microvesicles, and apoptotic bodies is based on differing biogenesis pathways, which confer variations in size, content, and surface protein composition [117]. Among these, exosomes are defined as the smallest EV subtype, with a diameter of approximately 30–150 nm. Their biogenesis is initiated by the invagination of the plasma membrane to form early endosomes. The subsequent inward budding of the endosomal membrane leads to the accumulation of intraluminal vesicles (ILVs), resulting in the formation of late endosomes, also known as multivesicular bodies (MVBs). Exosomes are then released into the extracellular space upon fusion of the MVB with the plasma membrane [118]. Following release, internalization by recipient cells can occur through multiple pathways, including endocytosis, membrane fusion, or receptor-mediated mechanisms. The term “exosome” was first coined in the 1980s following the observation of small vesicles, associated with the transferrin receptor, being released from sheep reticulocytes via receptor-mediated endocytosis and recycling [119,120].

The precise definition of exosomes remains a subject of ongoing debate. According to the recently updated Minimal Information for Studies of Extracellular Vesicles (MISEV2023) guidelines the term “extracellular vesicles” (EVs) broadly refers to particles that are naturally released from a cell, are delimited by a lipid bilayer, and cannot replicate [121]. More specifically, “exosomes” are defined as EVs of endosomal origin that are released upon the fusion of MVBs with the plasma membrane, whereas “ectosomes” (also referred to as microvesicles or microparticles) are generated by direct budding from the cell surface. However, due to the current limitations of isolation techniques in precisely distinguishing EVs based on their biogenetic pathways and the absence of definitive subtype-specific markers, the terms “exosomes” or “ectosomes” in much of the existing literature often refer broadly to mixed EV populations. It is critical to note that “small extracellular vesicles” (sEVs) and “exosomes” are not synonymous, as the former term encompasses both small ectosomes and exosomes. Given these persistent challenges in characterization and the lack of a universal marker, and our review’s focus on the therapeutic potential of exosomes as defined by size (30–150 nm) and endosomal origin, we retain “exosomes” as our core term. When discussing studies where the precise biogenesis is unclear, we use the more neutral term “EVs” to accurately reflect the cited literature. In contexts where we specifically refer to the 30–150 nm vesicle population under discussion, we use “exosomes” in line with our central theme.

### 3.2. Biological Characteristics and Functions of Exosomes

A distinctly enriched diversity of proteins and nucleic acids is observed in exosomes compared to their parent cells, indicating that cargo loading is an active and selective process which confers unique biological properties. While the mechanisms governing this selective incorporation are not fully elucidated, a critical role for the endosomal sorting complex required for transport (ESCRT) machinery in protein-specific sorting during MVB and ILV formation has been established [10]. Furthermore, involvement of tetraspanin proteins (e.g., CD9, CD63, CD81) and lipid-mediated interactions in cargo selection and exosome biogenesis have been demonstrated.

Following their release, internalization of exosomes by recipient cells occurs, enabling the regulation of a wide array of cellular behaviors. Exosomes have been implicated in vital physiological and pathological processes—including intercellular communication, immunoregulation, tissue repair and regeneration, and the progression of conditions such as cardiovascular disease—has been documented. Key biological processes influenced by exosomes include cell proliferation, migration, differentiation, and angiogenesis. Among these functions, intercellular communication is considered the most fundamental [13]. Through secretion into bodily fluids, exosomes facilitate the transport of functional cargo to both local and distant target cells. Subsequent regulation of extensive physiological and pathological processes, such as immune responses, tissue repair, and tumor metastasis, is achieved either through the activation of signaling pathways via receptor–ligand interactions or through the direct transfer of contents that alter the recipient cell’s state.

### 3.3. Exosome Isolation and Concentration

Prior to characterization and application, isolation and concentration of exosomes from biological fluids are typically required. The yield, purity, and integrity of the prepared exosomes are critical parameters, directly influencing the accuracy of experimental data and the clinical efficacy, stability, and biosafety of any resultant formulations [121]. Consequently, the development of efficient and precise purification methodologies is of paramount importance. However, given the heterogeneity of exosomes and the complexity of their source materials, no current technique achieves absolute purity. Existing methods primarily exploit specific biophysical properties—such as size, density, and surface composition—for separation, with each presenting distinct advantages and limitations. Therefore, selection of an appropriate method must be guided by the sample type, the required yield and purity for the intended application, and the nature of subsequent analyses. Several commonly employed techniques are summarized below [121,122,123].

#### 3.3.1. Differential Ultracentrifugation (DUC)

DUC remains the most widely adopted method for exosome isolation. This technique utilizes sequentially increasing centrifugal forces to pellet extracellular particles based on their differing sedimentation coefficients. While valued for its procedural simplicity and high yield, limitations in purity are associated with the method. Since separation is based on sedimentation rate rather than specific markers, co-precipitation of non-exosomal particles of similar size, such as protein aggregates, is common. Significant time and labor investments are also required [121,122,123].

#### 3.3.2. Density Gradient Ultracentrifugation (DGUC)

DGUC represents a refinement of the DUC approach, enhancing purity by separating particles based on their buoyant density within a medium such as sucrose or iopamidol. The cushioning effect provided by the gradient medium also helps preserve exosome integrity and biological activity. This improvement in purity, however, is achieved at the expense of a significantly reduced yield and necessitates extended processing time [121,122,123].

#### 3.3.3. Polymer Precipitation

Polymer precipitation is a straightforward technique that employs hydrophilic polymers, typically polyethylene glycol (PEG), to alter the solubility of exosomes, enabling their sedimentation at low centrifugal speeds. Numerous commercial kits based on this principle are available, offering advantages in operational simplicity, speed, and high yield. A major drawback is the concomitant precipitation of contaminants, including proteins, lipoproteins, and other vesicles, which compromises purity. The introduction of polymer residues into the final preparation may also interfere with downstream applications [123].

#### 3.3.4. Size Exclusion Chromatography (SEC)

SEC separates particles based on hydrodynamic volume by passing the sample through a porous stationary phase. Larger exosomes elute first, as they are excluded from the pores, while smaller soluble proteins are retained longer. This method is particularly suitable for viscous samples like plasma and serum, and it is renowned for preserving exosome biological activity and achieving high purity. Its primary limitation is a relatively low yield and sample dilution, often requiring a subsequent concentration step [121,123].

#### 3.3.5. Immunological Affinity Capture (IAC)

IAC achieves high specificity by utilizing antibodies against exosomal surface markers (e.g., tetraspanins CD9, CD63, CD81) immobilized on a solid support, such as magnetic beads. This allows for the highly selective isolation of exosome subpopulations bearing the target antigen. The primary disadvantage is that the bound antibodies are difficult to remove without compromising the exosome’s membrane integrity and biological function. Furthermore, the method is constrained by the availability and specificity of antibodies and cannot capture exosomes lacking the target epitope [122,123].

#### 3.3.6. Ultrafiltration

Ultrafiltration isolates exosomes based on size using membranes with defined molecular weight cut-offs (MWCO). It is one of the simplest and fastest techniques, available in charge-driven, centrifugal, and pressure-driven formats. A significant limitation is the potential for membrane fouling and clogging by exosomes and other particles, which can reduce recovery rates and reproducibility. Purity is also generally limited due to the non-specific nature of size-based filtration [122,123].

#### 3.3.7. Other Methods

Microfluidic technology has emerged as a powerful tool for rapid, efficient exosome separation, leveraging principles of size or immunoaffinity within miniaturized devices [124]. This approach offers potential for automation and high-throughput analysis but is currently limited by specialized equipment requirements and a lack of standardization and scalability. Additional methods, including ion-exchange chromatography and various affinity probes, have been developed based on surface charge or other molecular interactions [121]. Furthermore, for research purposes, the transfection of parent cells with fluorescently labeled exosome markers enables in vivo tracing and visualization, providing valuable tools for elucidating exosome fate and function in physiological and pathological processes [125].

## 4. Therapeutic Effects of Exosomes on Dry Eye Disease

### 4.1. Non-Sjögren’s Syndrome-Related Dry Eye Disease

DED is a multifactorial, symptomatic condition characterized by a loss of tear film and ocular surface homeostasis, with key etiological factors including tear film instability, hyperosmolarity, ocular surface inflammation, and neurosensory abnormalities [126]. These pathophysiological disturbances lead to characteristic symptoms such as gritty sensation, burning, and blurred vision [127]. The global prevalence of DED is estimated to range from 5% to 50% [4]. Based on etiology, DED is primarily categorized into aqueous-deficient dry eye (ADDE) and evaporative dry eye (EDE) [128], with ADDE further subdivided into SS-associated and non-SS-associated DED [129].

Despite available pharmacological treatments—including artificial tears, anti-inflammatory agents, and immunosuppressants [130,131]—no single therapy achieves a complete cure, a shortfall attributed to the disease’s complex pathophysiology. In recent years, exosomes have emerged as a promising novel therapeutic, demonstrating significant potential for modulating immunity, inflammation, oxidative stress, and repair/regeneration processes in DED [69,78]. Autologous serum eye drops, initially developed for patients with corneal alkali burns, are now widely used to treat DED and other ocular surface diseases. Due to its pH and osmolarity similar to natural tears, autologous serum serves as an ideal alternative to artificial tears. The vitamins, growth factors, and proteins it contains are known to support corneal epithelial function. However, the clinical efficacy of autologous serum therapy exhibits considerable inter-individual variability. In contrast, exosomes demonstrate more stable and predictable therapeutic effects in moderate-to-severe ocular surface diseases, attributed to their lower levels of pro-inflammatory cytokines [40]. Platelet-rich plasma, another blood-derived product, shares similar characteristics with autologous serum. Compared to platelet-rich plasma, exosomes contain fewer free proteins, which theoretically reduces variability associated with different production devices and offers greater potential for standardization [52]. Cyclosporine A eye drops and lifitegrast, both representative anti-inflammatory and immunosuppressive agents widely used in clinical practice, offer well-defined mechanisms of action, rapid onset, and favorable safety profiles. Unlike these single-target agents, exosomes exert multi-target effects, which may provide more pronounced therapeutic potential—including the ability to reverse pathological changes—in complex multifactorial diseases such as DED [132]. Emerging biologic therapies, exemplified by JAK inhibitors and targeted monoclonal antibodies, represent an evolution from conventional anti-inflammatory approaches toward precision immunomodulation. These agents may form a complementary relationship with the multi-target natural mechanisms of exosomes.

Although the pathogenesis of DED remains incompletely elucidated, ocular surface immune and inflammatory responses are recognized as pivotal components of its vicious cycle. Environmental stressors such as tear film instability and hyperosmolarity stimulate ocular surface epithelial cells to release inflammatory signaling molecules [133], including damage-associated molecular patterns (DAMPs), reactive oxygen species (ROS), and matrix metalloproteinases (MMPs) [134], thereby initiating inflammatory cascades. Among these, ROS are of particular significance, causing oxidative stress, lipid peroxidation, altered membrane permeability, and DNA damage, which collectively lead to extensive ocular surface injury. Notably, ROS not only regulate the initiation of the inflammatory cycle but also exacerbate each subsequent stage [135], representing a major factor limiting the efficacy of conventional anti-inflammatory therapies. To address ROS-mediated damage, various exosome-based antioxidant strategies have been developed. For instance, a novel therapeutic nanoparticle was developed by growing cerium oxide nanocrystals in situ on MSC-derived exosomes (MSC-Exo@Ce) [73]. This formulation was demonstrated to effectively scavenge ROS, suppress inflammation, and promote corneal cell proliferation in vitro and in vivo, combining the regenerative properties of exosomes with the antioxidant capacity of cerium while exhibiting excellent biocompatibility. Similarly, a multifunctional eyedrop was prepared through the in situ deposition of ascorbic acid-reduced gold nanoparticles onto MSC-derived exosomal membranes (MSC-Exo@AA) [69]. In a mouse DED model, MSC-Exo@AA demonstrated superior efficacy compared to its individual components in promoting corneal epithelial repair, reducing inflammation, lowering ROS levels, and increasing tear secretion, while maintaining excellent biosafety.

The NLRP3/IL-1β signaling axis represents a key pathway through which ROS exacerbates the inflammatory process. Hyperosmotic stress induces ROS production, which acts as an initiating signal for the downstream NLRP3-caspase-1 pathway. This subsequently activates the mitogen-activated protein kinase (MAPK) and nuclear factor kappa B (NF-κB) pathways, promoting the secretion of IL-1β and IL-18 and intensifying ocular surface inflammation [136]. Elevated expression of NLRP3 inflammasomes and associated mediators, including caspase-1, IL-1β, and IL-18, has been confirmed in both DED patients and animal models [137,138]. The suppression of this pathway has been demonstrated using human adipose-derived stem cell-derived EVs (hADSC-EVs), which inhibit NLRP3 inflammasome activation and IL-1β secretion in DED models [79]. These findings were further corroborated by Wang et al. [74], who showed that mouse adipose-derived stem cell-derived exosomes (mADSC-Exos) downregulate the expression of NLRP3, caspase-1, IL-1β, and IL-18 in the conjunctiva of DED mice, thereby promoting ocular surface epithelial repair, restoring goblet cell function, and increasing tear secretion.

Further inflammatory signaling in DED is propagated through the activation of the MAPK/NF-κB pathway [75]. Sequencing of miRNAs in human umbilical cord MSC-derived EVs (hUCMSC-EVs) identified the top 10 immunity-related miRNAs, among which miR-125b, let-7b, and miR-6873—conserved between humans and mice—were associated with the activated IRAK1/TAB2/NF-κB pathway in DED [75]. Furthermore, bone marrow-derived MSC-derived exosomes (BMSC-Exos) were shown to suppress TLR4/MyD88/NF-κB signaling by delivering miR-21-5p to CD4^+^ T cells, thereby modulating the regulatory T cells (Treg)/T helper 17 cells (Th17) balance to alleviate DED in mice [80]. However, given the extensive and complex role of NF-κB in immune responses [139], its direct targeting may induce adverse effects. To circumvent this, an engineered approach was developed in which siRNA targeting the NFKBIZ gene was fused with exosomes to form anti-NFKBIZ siRNA HEV constructs [76]. These constructs retained the innate targeting capability of exosomes and significantly reduced the secretion of ocular surface inflammatory cytokines through efficient NFKBIZ knockout in target cells, demonstrating potent and specific anti-inflammatory properties.

The inflammatory milieu of DED promotes the maturation of antigen-presenting cells (APCs), particularly dendritic cells (DCs), which initiates adaptive immune responses, and drives T cell polarization toward Th1 and Th17 pathways. Subsequent migration of effector T cells to the conjunctiva results in the secretion of IFN-γ, IL-17, and other inflammatory cytokines, which trigger inflammatory cascades that further exacerbate ocular surface damage [140]. Notably, treatment with MSC-EVs has been shown to attenuate DC recruitment and maturation, as well as suppress Th17 cell-mediated immune responses, both in human corneal epithelial cells (HCECs) under hypertonic stress and in mouse models [67]. This indicates that MSC-EVs suppress ocular surface inflammation by inhibiting DC-mediated Th17 immune responses.

The fate of inflamed tissues is largely governed by the balance between pro-inflammatory M1 and anti-inflammatory M2 macrophage polarization. Induction of the M1 state is driven by Th1-associated cytokines, leading to the production of proinflammatory mediators, while a shift toward the M2 phenotype is promoted by Th2-associated cytokines, which secrete anti-inflammatory factors to suppress inflammation and maintain immune homeostasis [141]. During DED, close interaction occurs between macrophages and infiltrating CD4^+^ T cells in the conjunctiva [142]. Reprogramming of proinflammatory M1 macrophages into an anti-inflammatory M2 phenotype by MSC-Exos has been demonstrated, a process mediated by miR-204 targeting of the IL-6/IL-6R/Stat3 pathway, which restores ocular surface immune homeostasis in mouse and human GVHD-associated DED [81]. Furthermore, M2 macrophage-derived EV (M2-EV) treatment was found to more effectively maintain ocular surface homeostasis and alleviate symptoms in a DED mouse model compared to hyaluronic acid (HA), fungal metabolite (FM), or M0-EV treatments, while also significantly reducing proinflammatory factors such as IL-1β [77]. Macrophage polarization was also influenced by engineered exosomes; constructs formed by fusing siRNA against the NF-κB IZ gene with exosomes (anti-NF-κB IZ siRNA HEV) were shown to reduce ocular surface inflammatory cytokines and polarize infiltrating macrophages from an M1 to an M2 phenotype [76].

The role of conjunctival goblet cells (CGCs) in mucosal immunity is also significant. Antigens can bind to mucins secreted by CGCs and be channeled to adjacent phagocytes, particularly CD11b^+^ F4/80^+^ macrophages, via CGC-associated channels, with CGC loss being correlated with abnormal M1 macrophage polarization [143]. Protection of CGCs from M1 macrophage-mediated inflammation by exosomes has been observed. Specifically, exposure of CGCs to supernatant from M1 macrophages that had been treated with periodontal ligament stem cell exosomes (PDLSC-Exos) was shown to significantly enhance Muc5ac expression under cholinergic stimulation [71], indicating that PDLSC-Exos can protect CGC function.

Beyond immunomodulation, therapeutic effects are also exerted by exosomes through other pathways, such as specific miRNA delivery. Mitigation of ocular surface damage and inflammation in mouse DED models was achieved by exosomes derived from mADSCs through the delivery of miR-233-3p, which inhibits the E3 ubiquitin ligase F-box and WD repeat domain-containing 7 (Fbxw7) [144]. Similarly, alleviation of inflammation and apoptosis in hyperosmotic-induced HCECs and a benzalkonium chloride (BAC)-induced DED mouse model was demonstrated for hUCMSC-Exos, which target the protein SQSTM1 via miR-146a [65]. The potential role of endogenous exosomes is also under investigation; enrichment of inflammation-associated miRNAs was identified in tear EVs from DED patients via RNA sequencing, suggesting participation of tear exosomes and their miRNA cargo in DED pathogenesis, though their specific origins and functions require further validation [70].

**Figure 2:**
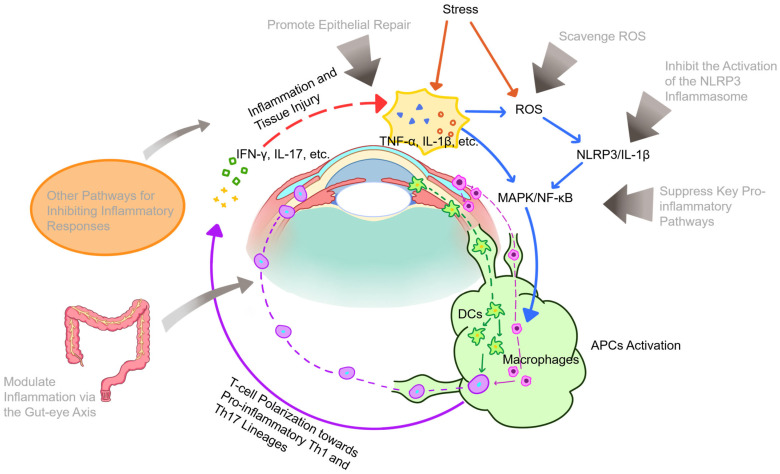
The Vicious Cycle of DED and the Multi-Targeted Therapeutic Role of Exosomes. This schematic illustrates the self-perpetuating pathophysiological cycle of DED. The cycle is initiated by etiological factors such as tear film instability and hyperosmolarity, which cause ocular surface stress and damage. This stress triggers the release of ROS and inflammatory mediators, leading to the activation of key pro-inflammatory signaling pathways, including the NLRP3/IL-1β axis and MAPK/NF-κB. This inflammatory milieu promotes the maturation of APCs like DCs, macrophages are polarized to the M1 phenotype, driving T-cell polarization towards pro-inflammatory Th1 and Th17 lineages, further amplifying inflammation and tissue injury. These events lead to corneal/conjunctival epithelial damage, goblet cell loss, and neurosensory abnormalities, which in turn exacerbate the initial tear film instability, thus closing the vicious cycle. Exosomes intervene at multiple critical points to break this cycle through mechanisms including antioxidant effects (e.g., MSC-Exo@Ce, MSC-Exo@AA scavenging ROS), inflammasome suppression (inhibiting NLRP3 activation and IL-1β/IL-18 secretion), signaling pathway inhibition (suppressing NF-κB/MAPK via miRNAs such as miR-21-5p and miR-125b), immune cell modulation (reducing DC maturation, inhibiting Th17 responses, and promoting M1-to-M2 macrophage polarization via miR-204), tissue repair and protection (enhancing epithelial repair, goblet cell function, and Muc5ac expression, and delivering regenerative miRNAs like miR-146a and miR-233-3p), and systemic modulation (probiotic-derived exosomes regulating inflammation via the gut–eye axis). DED, dry eye disease. ROS, reactive oxygen species. APCs, antigen-presenting cells. DCs, dendritic cells.

Finally, a systemic pathway involving gut–eye axis communication is implicated in exosome-mediated therapy. Improvement of DED phenotypes in mouse models was observed following administration of *Lactobacillus fermentum* HY7302, which regulated pro-inflammatory and apoptotic factor expression [145]. Subsequent in vitro experiments using exosomes isolated from this probiotic revealed a significant reduction in pro-inflammatory cytokine gene expression in BAC-treated human conjunctival cells, alongside an increase in tight junction protein gene expression in Caco-2 intestinal cells [68]. Furthermore, a reduction in proinflammatory cytokine expression was observed when these exosomes were added to a Transwell co-culture system of Caco-2 cells and conjunctival epithelial cells, demonstrating a therapeutic effect mediated by probiotic exosomes via the gut–eye axis [68] (Figure 2).

### 4.2. Dry Eye Disease Associated with Sjögren’s Syndrome

In SS-associated DED, the immunomodulatory properties of exosomes are particularly prominent. The disease is characterized by lymphocytic infiltration of exocrine glands, notably the lacrimal glands, where CD4^+^ T cell-mediated autoimmune responses are pivotal. Pathogenesis is centrally driven by an imbalance between Treg and Th17 [146,147,148,149], with autophagy serving as a key mechanism regulating T cell homeostasis [150]. Analysis of peripheral blood lymphocyte subsets in primary SS (pSS) patients revealed that exosomes derived from hUCMSCs suppress abnormal CD4^+^ T cell proliferation and apoptosis through the inhibition of autophagy, thereby restoring the Th17/Treg balance [109]. Similarly, amelioration of SS in mouse models was demonstrated ADSC-Exos via modulation of the Th17/Treg balance [151]. This effect was further enhanced by transfecting adipose-derived exosomes with miRNA let-7f-5p, which was shown to improve SS in mice by inhibiting Th17 cells through targeted suppression of the RORC/IL-17A signaling axis [112]. Conversely, exacerbation of pSS immunopathology has been linked to endogenous EVs; upregulation of miR-501-3p was identified in plasma EVs from pSS patients, where it promotes CD4^+^ T cell activation and differentiation into Th1 and T follicular helper (Tfh) cells [108]. Furthermore, evidence for T cell-driven pathogenesis via the exosomal pathway was provided by the finding that activated T cells secrete exosomes containing miR-142-3p, which subsequently modulate glandular cell function [105].

A crucial role for macrophages in the development and resolution of SS-associated DED inflammation is also supported by existing evidence [142]. Promotion of macrophage polarization toward the anti-inflammatory M2 phenotype and Treg generation by hUCMSC-Exos has been demonstrated, a process mediated via miR-100-5p that alleviates SS-induced dacryoadenitis [107]. Similar to non-SS DED, the involvement of the MAPK/NF-κB pathways is observed in SS. EVs from induced pluripotent stem cells (iPSC-EVs) were found to contain high levels of let-7 family miRNAs and to suppress TLR4 and NF-κB expression, thereby inhibiting MAPK-mediated proinflammatory cytokine production [110].

The pathogenesis of SS is also closely correlated with gut dysbiosis, the severity of which is associated with both ocular and systemic manifestations [152]. Modulation of gut microbiota and Treg/Th17 cell immunity by UCMSC-Exos was observed following the in vitro co-culture and subsequent reinfusion of non-obese diabetic (NOD) mouse splenic T cells, resulting in improved disease phenotypes [114]. However, as this model did not involve direct exosome administration in vivo, further investigation is required to confirm a direct causal relationship.

Finally, the role of myeloid-derived suppressor cells (MDSCs) is of growing interest. MDSCs represent a heterogeneous population of immature myeloid cells with broad immunosuppressive capabilities, interfering with the activation of T cells, B cells, and natural killer cells while promoting Treg induction to modulate innate and adaptive immunity [153,154]. A gradual diminishment of this immunosuppressive function is observed as SS progresses [155]. Enhancement of MDSC function by exosomes has been demonstrated; olfactory ecto-MSC-derived exosomes (OE-MSC-Exos) were shown to activate the Jak2/Stat3 pathway in MDSCs via IL-673. These exosomes were also found to be enriched with S100A4, which promotes IL-6 production in MDSCs through the TLR4 pathway, thereby creating a positive feedback loop that enhances immunosuppressive capacity [111]. A further regulatory role for MDSCs in SS is suggested by the finding that they can suppress germinal center B cells through EV-mediated delivery of miR-10a-5p, which targets Bcl-6 [113].

## 5. Therapeutic Effects of Exosomes on Corneal Injury

The cornea, situated at the eye’s anterior pole, functions as a highly precise and transparent light-transmitting structure. Its direct exposure to the external environment, however, renders it vulnerable to injuries from mechanical trauma, radiation, chemical irritants, and temperature extremes. The healing process following corneal injury, categorized by anatomical layer, involves the repair of the epithelium, stroma, and endothelium. These processes, while distinct in their characteristics, are interconnected and mediated by various growth factors, cytokines, and extracellular matrix (ECM) remodeling.

### 5.1. Corneal Epithelial Injury

Corneal epithelial wound healing is a multistage process involving cellular reorganization, epithelial cell migration from the wound margin, proliferation of limbal and peripheral cells, and subsequent adhesion, differentiation, and stratification to restore the normal epithelial structure [156]. Exosomes from diverse sources have been demonstrated to promote this healing by enhancing epithelial cell proliferation, migration, and differentiation, while concurrently modulating immune responses to suppress local inflammation. For instance, acceleration of corneal epithelial repair by BMSC-EVs was shown to occur through the boosting of cell proliferation and inhibition of CASPASE-3-mediated apoptosis in vitro [44]. Similarly, promotion of HCECs proliferation and migration was achieved by BMSC-Exos via activation of the p44/42 MAPK pathway in vitro, while inflammation, fibrosis, and neovascularization were inhibited in vivo in an alkaline burn model [62].

The critical role of EVs is further underscored by the finding that EV-depleted BMSCs exhibit a significantly reduced capacity for corneal wound healing, with the therapeutic effect being dose-dependent [17]. This reparative and anti-inflammatory potential has been confirmed using advanced in vitro models that simulate ocular surface injury with human corneal structures and microfluidic technology [59], as well as in ex vivo models using 1-heptanol-injured corneas [18]. The therapeutic profile of exosomes can also be optimized; exosomes generated from BMSCs cultured within GelMA hydrogels (3D-Exos) were shown to exert enhanced anti-inflammatory, pro-proliferative, and tissue remodeling effects by delivering miR-150-5p to target the *PDCD4* gene [55]. Furthermore, a unique anti-inflammatory phenotype was observed in exosomes derived from TGF-β1-preconditioned BMSCs, which demonstrated superior efficacy in an alkali burn model compared to exosomes from untreated or IFN-γ-preconditioned cells [20].

Beyond BMSCs, exosomes from other sources show promise. Enhancement of corneal epithelial cell migration by ADSC-Exos has been demonstrated both in vivo and in vitro [32]. As an alternative to autologous serum, which has variable outcomes due to its complex and potentially pro-inflammatory composition, serum-derived EVs have been applied to the ocular surface, demonstrating effective wound-healing properties with a reduced risk of inducing inflammation [40]. Repair of mouse corneal cryoinjuries has also been achieved using mouse amniotic fluid-derived MSC exosomes (mAF-MSC-Exos), which act by releasing DNMT1 to downregulate miR-33 and upregulate Bcl6 expression in corneal epithelial cells [54]. Even salivary-derived exosomes have been shown to promote wound healing by enhancing migration, proliferation, and mitochondrial function in HCECs and human limbal epithelial stem cells (LESCs) [30].

Given the ectodermal origin of the cornea, exosomes from ectoderm-derived cells are of particular interest. Human induced pluripotent stem cell-derived retinal organoid exosomes (iPSC-RO-Exos) were demonstrated to promote corneal epithelial wound healing in vivo by increasing proliferation, suppressing inflammation, and regulating wound-healing associated substances like retinoic acid [28]. A superior effect on HCEC proliferation, migration, cell cycle progression, and apoptosis inhibition was exhibited by iPSC/MSC-Exos compared to MSC-derived exosomes alone, driven by the upregulation of cyclin A and CDK2 [48]. This suggests that therapeutic efficacy may be correlated with cellular pluripotency and developmental origin, as BMSC-EVs have also been shown to possess stronger epithelial repair and anti-inflammatory effects than EVs derived from HCECs [45].

The natural double-layered membrane structure of exosomes also renders them ideal drug delivery vehicles. Superior efficacy in treating corneal epithelial damage has been demonstrated by strategies that combine exosomes with other active ingredients, leveraging their targeting capability and stability. For example, synergistic effects were observed when anti-TNF-α antibodies were conjugated to the surface of ADSCs for treating alkaline burns [60]. Similarly, incorporation of dexamethasone into milk-derived exosomes enabled targeted delivery to macrophages, modulating the Wnt signaling pathway to suppress inflammation and promote healing in alkaline burns [50]. Transfection of miR-29b-3p into BMSC-Exos was shown to activate autophagy by inhibiting the PI3K/AKT/mTOR pathway, thereby suppressing inflammation and reducing fibrosis [31]. To prolong residence time, BMSC-EVs have been resuspended in methylcellulose, creating a formulation that modulated cell death, inflammation, and angiogenesis in injured tissue without being internalized by intact corneas, thus minimizing side-effect risks [38]. A thermosensitive hydrogel delivery system for miR-24-3p-transfected ADSC-Exos has also been developed, significantly promoting epithelial migration while reducing stromal fibrosis and macrophage activation [42].

The role of oxygen and specific proteins in exosome-mediated healing is also being elucidated. Thrombospondin-1 (TSP-1), a key factor present in corneal stromal and endothelial cells, participates in chemically induced wound healing by exerting anti-inflammatory and anti-angiogenic effects. Improvement of hypoxia-induced paracellular leakage in HCECs and promotion of wound healing by TSP-1 were demonstrated to occur through the regulation of exosomal protein expression, identifying a novel therapeutic target for hypoxic corneal lesions [157]. Finally, an innovative approach involved the creation of oxygen-containing exosomes coated with hemoglobin nanoparticles (OExo-NPs), which significantly promoted HCEC proliferation and migration, effectively alleviated hypoxia, and inhibited angiogenesis, inflammation, and scar formation [25].

### 5.2. Corneal Stromal Injury

The corneal stromal wound healing process is initiated by rapid apoptosis and necrosis of keratocytes adjacent to the injury site. Subsequent proliferation, migration, and activation of surrounding reserve keratocytes into fibroblasts are driven by factors such as TGF-β, with a subset of these fibroblasts further differentiating into α-SMA-expressing myofibroblasts that mediate wound contraction. A temporary ECM scaffold is secreted by these activated cells, while the concomitant release of chemokines triggers inflammatory cell infiltration. Collective participation in matrix repair and remodeling is mediated by collagenolytic proteases secreted by all involved cell types. This complex process is frequently complicated by corneal scarring, opacity, stromal fibrosis, and visual impairment. Ultimately, the restoration of normal stromal structure and function is dependent upon the resorption of abnormal ECM and the apoptosis or phenotypic reversal of myofibroblasts [156].

As previously indicated, the application of exosomes has been demonstrated to reduce fibrosis and scar formation in the stroma. For instance, the prevention of oxidative damage and inhibition of corneal stromal cell apoptosis by ADSC-Exo eye drops were achieved through the upregulation of Bax and downregulation of Bcl2, thereby suppressing α-SMA expression, reducing scar formation, and promoting healing in a rat model [32]. Furthermore, the inhibition of corneal neovascularization is critical for preserving transparency post-injury. MMPs, a large family of zinc-dependent endopeptidases, are essential for ECM remodeling. Specifically, MMP14 is upregulated in the corneal stroma during wound healing and neovascularization. Transport of proteins, including MMP14, to vascular endothelial cells by exosomes secreted from corneal fibroblasts has been demonstrated [22]. MMP14 was further shown to alter exosomal levels of MMP2 and significantly influence its transport. A potential mechanism for influencing corneal neovascularization by MMP14-containing exosomes was identified as the degradation of vascular endothelial growth factor receptor 1 (VEGFR1) and its ligand VEGFA, thereby inhibiting endothelial cell proliferation and migration [24]. An alternative strategy involved the combination of platelet-derived EVs (PEVs) with the anti-angiogenic agent kaempferol to form PEV-KM, which was efficiently internalized by human vascular endothelial cells, downregulating angiogenesis-related genes and significantly inhibiting neovascularization in an alkali-induced mouse model while reducing pro-angiogenic and inflammatory cytokines [102]. Similarly, inhibition of angiogenesis was achieved by incorporating fluorescein amide (FAM)-labeled VEGFA siRNA into exosomes, which targeted human vascular endothelial cells and induced cell death by reducing VEGFA/PIGF/VEGFC levels upon degradation [101].

### 5.3. Corneal Endothelial Injury

Human corneal endothelial cells (HCEnCs) possess limited proliferative capacity in vivo, being predominantly arrested in the G1 phase of the cell cycle, a state closely associated with contact inhibition. Consequently, corneal endothelial wound healing occurs primarily through cell migration and spreading, during which HCEnCs enlarge and lose their characteristic hexagonal morphology. A transient acquisition of a fibroblast-like morphology, termed endothelial–mesenchymal transition, is also observed during this process [156]. A significant risk of chronic, irreversible corneal edema arises when HCEnC density falls below a critical threshold of 500 cells/mm^2^ [158]. Pathological acceleration of HCEnCs loss can be caused by viral infections, inflammation, surgical trauma, and corneal endothelial dystrophies, with Fuchs’ endothelial corneal dystrophy (FECD) being a common etiology [36].

The role of endogenous EVs appears complex. Inhibition of cell proliferation, promotion of apoptosis, and increased cell volume were observed following the treatment of the HCEnC-12 cell line and primary HCEnCs with EVs derived from HCEnCs [82]. Furthermore, impairment of wound healing capacity was demonstrated in vitro and in ex vivo models using human, porcine, and rabbit corneal endothelia, suggesting that the uptake of EVs by corneal endothelial cells may intrinsically limit their self-renewal [82]. Further investigation revealed that proliferation capacity in HCEnCs and FECD cells is restricted by miR-195-5p expression. Promotion of HCEnCs proliferation was achieved by inhibiting miR-195-5p via anti-miRNA therapy, opening potential avenues for in vivo treatments to delay FECD progression [83].

In contrast, a therapeutic benefit is offered by exogenously applied exosomes. Promotion of wound healing and endothelial cell regeneration by ADSC-Exos was demonstrated through the induction of cell cycle transition and suppression of senescence and autophagy [37]. A proliferative effect on damaged HCEnCs was also observed with MSC-EVs [35]. Anti-inflammatory and regenerative effects in LPS-induced HCEnC injury models were exhibited by M2a macrophage-derived exosomes, with these properties being enhanced by epidermal growth factor (EGF) pretreatment, suggesting a potential therapeutic approach for inflammatory endothelial conditions [29]. Isolation and analysis of PEVs revealed a mixture of growth factors, nutrients, and proteins associated with cellular adhesion pathways [29]. Higher viability, increased wound healing rates, stronger proliferation markers, and enhanced adhesion were observed in HCEnCs treated with PEVs, without induced cytotoxicity [52].

Given that endoplasmic reticulum (ER) stress is a primary mechanism in endothelial dystrophy, leading to apoptosis and cell loss, its modulation is a key therapeutic target. Significant downregulation of most ER stress-related genes in HCEnCs under stress conditions was achieved by MSC-EVs [52]. Concurrently, upregulation of the Akt signaling pathway, inhibition of caspase-3 activation, and a reduction in apoptosis were observed. This protective effect was associated with the delivery of multiple ER stress-targeted miRNAs to the corneal endothelial cells [15]. In contrast, serum-EVs were found to primarily induce Akt phosphorylation with limited effects on ER stress regulation and apoptosis inhibition, suggesting that MSC-EVs possess a unique and potent therapeutic profile for ER stress-related corneal endothelial disorders [15].

### 5.4. Corneal Injury and Intercellular Communication

Crucial regulatory roles in corneal wound healing are played by intercellular communication among the corneal epithelium, stroma, and endothelium. Cytokines secreted by epithelial cells, such as TGF-β1 and PDGF, can traverse a disrupted basement membrane to induce the differentiation of stromal fibroblasts into myofibroblasts [159]. Subsequent production of growth factors like HGF by these myofibroblasts then provides feedback that promotes corneal epithelial cell proliferation [160]. Concurrently, FGF-2 secretion by epithelial cells during wound healing can trigger endothelial–mesenchymal transition [161]. As understanding of exosome signaling deepens, significant attention has been directed toward their role in facilitating this inter-structural communication within the cornea.

Bidirectional communication between the corneal epithelium and stroma is particularly critical for wound repair. Proteomic analysis of EVs isolated from human corneal keratocytes (HCKs), fibroblasts (HCFs), and myofibroblasts (HCMs) revealed that HCM-EVs possess a distinct protein cargo and significantly promote the migration, proliferation, and motility of HCECs more effectively than EVs from HCKs or HCFs [57]. This enhanced effect may be related to the expression of factors such as CXCL1, CXCL6, CXCL12, MMP1, or MMP2 by HCM-EVs, though the precise mechanisms require further validation. Furthermore, induction of myofibroblastic transformation in corneal fibroblasts has been demonstrated following fusion with mouse CEC-Exos in vitro; these exosomes were also shown to promote endothelial cell proliferation and sprouting, thereby participating in both wound healing and neovascularization [23]. The capacity of EVs to penetrate the posterior elastic layer and mediate communication between stromal and endothelial cells has also been documented [162]. Acceleration of wound healing in scratched HCECs in vitro has been consistently demonstrated using exosomes derived from HCECs, HCFs, and HCEnCs [19].

### 5.5. Limbal Stem Cell Injury and Regeneration

The limbal niche is a specialized microenvironment composed of LESCs, corneal stromal stem cells (CSSCs), limbal melanocytes (LMs), and a unique ECM [163]. Continuous renewal of the corneal epithelium is driven by LESCs, whose function and pluripotency are intrinsically maintained by this niche. A symbiotic relationship exists between LESCs and other niche cells like CSSCs, mediated not only by direct cell–cell contact but also crucially by paracrine mechanisms involving soluble factors and exosomes. Comparison of the protein cargo in exosomes derived from LESCs, CSSCs, and LMs revealed that LESC-Exos are uniquely enriched with factors associated with keratinocyte development, extracellular matrix organization, and niche regulation [164]. Promotion of LESC proliferation and stemness maintenance by CSSC-derived EVs has been demonstrated, a process mediated by miR-663b targeting of the Notch signaling pathway [100]. Uptake of exosomes from CSSCs and LMs by LESCs has been confirmed, with proteomic analysis indicating their participation in signaling pathways related to extracellular matrix deposition and intercellular communication [103]. Specifically, proteins involved in collagen remodeling and cell–matrix adhesion were identified in CSSC-Exos, while LM-Exo proteins were more associated with melanosome distribution and oxidative stress processes. A role for LESCs in promoting CSSC proliferation and maintaining stemness has also been suggested by the finding that LESC-Exo-treated CSSCs exhibit reduced keratinocyte marker expression, increased MSC marker expression, and raised proliferation rates [46].

Damage to LESCs and their niche from chemical burns, prolonged contact lens wear, or chronic inflammation can lead to limbal stem cell deficiency (LSCD) [165]. This condition is characterized by severe corneal pathologies, including persistent epithelial defects, stromal neovascularization, conjunctival epithelial invasion, and ultimately corneal opacification and vision loss. Induction of transdifferentiation tendencies between corneal and conjunctival epithelial cells has been observed via EV-mediated signaling, accompanied by a dedifferentiation intermediate state [99]. This suggests that the conjunctivalization process in LSCD may be associated with EV-mediated signaling, potentially involving miR-9-5p modulation of HES-1 to regulate stem cell homeostasis and differentiation. In a therapeutic context, suppression of postoperative corneal neovascularization is achieved when cultured oral mucosal epithelial cells (COMECs) for transplantation are co-cultured with limbal niche cells (LNCs) as a feeder layer. High-throughput sequencing revealed that exosome-mediated intercellular signaling is a key factor in this LNC-driven angiogenesis inhibition [97]. Enhancement of LESC colony formation capacity and epithelial–mesenchymal transition (EMT) by ADSC-EVs has been demonstrated, implying maintenance of LESC stemness, with miR-25, miR-191, and miR-335 identified as the most likely functional miRNA factors [98]. Similarly, enhancement of hLESC migration, proliferation, and mitochondrial function has been demonstrated using salivary exosomes [30]. The clinical potential of this approach is supported by a study in which satisfactory corneal epithelial regeneration and limbal repair progression were observed in cats with LSCD following 30 days of treatment with feline ADSC-Exo eye drops, outperforming dexamethasone treatment [96].

**Figure 3:**
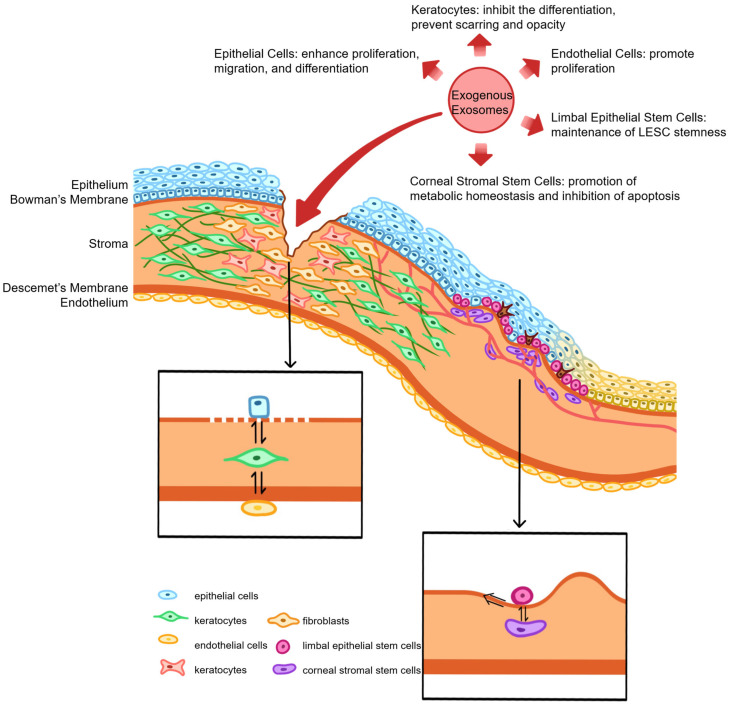
Mechanisms of Corneal Wound Healing and the Communicative and Therapeutic Role of Exosomes. This schematic illustrates the complex process of corneal wound healing across its three main layers—epithelium, stroma, and endothelium—and highlights the pivotal role of exosomes in facilitating intercellular communication and providing therapeutic intervention. A key feature of corneal repair is the bidirectional crosstalk between layers, mediated by endogenous exosomes. Epithelial cell-derived exosomes can influence stromal fibroblasts, while stromal cell-derived exosomes promote epithelial cell migration and proliferation. Exosomes can also traverse the posterior elastic layer to mediate communication between the stroma and endothelium. Furthermore, within the limbal niche, exosomes from limbal epithelial stem cells, corneal stromal stem cells, and limbal melanocytes reciprocally regulate each other’s proliferation, stemness, and niche homeostasis. Therapeutically, exogenously applied exosomes from various sources target specific pathological processes in each layer: in the epithelium, they enhance proliferation, migration, and differentiation while suppressing apoptosis and inflammation; in the stroma, they inhibit fibroblast-to-myofibroblast differentiation, reduce collagen deposition, suppress neutrophil infiltration, prevent scarring, and inhibit neovascularization; in the endothelium, they promote proliferation by suppressing senescence, autophagy, and ER stress-induced apoptosis; and in the limbal niche, they enhance stem cell colony formation, migration, and stemness maintenance. Advanced delivery strategies, such as embedding exosomes in hydrogels or conjugating them with drugs/siRNA, further enhance stability, retention, and targeted efficacy, collectively promoting the restoration of corneal transparency and function.

Exosomes derived from CSSCs have also been shown to prevent fibrotic scar formation and stimulate the regeneration of transparent stromal tissue after corneal injury in mice, a mechanism closely associated with the blocking of neutrophil infiltration [41]. A reduction in fibrosis gene expression (Col3a1, Acta2), suppression of neutrophil infiltration, and restoration of normal tissue morphology were demonstrated following CSSC-Exo treatment. These effects were abolished by Alix knockdown, which significantly decreased exosomal miRNA content, suggesting reliance on Alix-dependent miRNA delivery [41]. Further in vivo and in vitro experiments confirmed that a reduction in corneal inflammation and fibrosis by hCSSC-EVs is achieved through the expression of miR-29a, mediating the anti-scarring effect [56]. Enhancement of anti-fibrotic and pro-regenerative capabilities has also been observed; pretreatment of hCSSCs with melatonin yielded exosomes that upregulate TGFβ3 and PPARγ while downregulating TGFβ1 expression in immature hCSSCs [16]. Finally, given the participation of ER stress and the unfolded protein response (UPR) in ocular pathogenesis, the promotion of metabolic homeostasis and inhibition of apoptosis in CSSCs under ER stress conditions by ADSC-EVs represents a significant protective mechanism [34] (Figure 3).

### 5.6. Diabetes-Related Corneal Pathology

With the rising global prevalence of diabetes mellitus (DM), diabetic corneal disease is being encountered with increasing frequency. Studies suggest that 47–64% of diabetic patients may be affected by varying degrees of primary diabetic keratopathy (DK) [166,167]. Clinical manifestations primarily include DED, altered corneal sensitivity, delayed epithelial wound healing following surgery or trauma, and neurotrophic corneal ulcers. The underlying pathological mechanisms are multifactorial, potentially involving stem cell dysfunction, corneal epithelial and endothelial abnormalities, basement membrane alterations, advanced glycation end-product deposition, corneal neuropathy, oxidative stress, and inflammatory responses [166,167]. Delayed corneal wound healing in diabetes has been specifically associated with irregular fibrin expression, persistent inflammatory responses, and neuropathic defects [168]. Given that nearly half of all diabetic patients exhibit increased susceptibility to injury and infection alongside impaired epithelial healing [169], the development of strategies to improve corneal repair in this population is of critical importance.

Alterations in exosomal expression profiles under diabetic conditions have been documented. Comparison of exosomes derived from LESCs of diabetic and non-diabetic origins revealed distinct miRNA and protein cargos, with exosomes from non-diabetic LESCs inducing higher proliferation rates in CSSCs [46]. This suggests that compositional differences in diabetic exosomes may contribute to the disease phenotype. Clinical analysis has further indicated significantly reduced expression of Flotillin-2 protein in plasma exosomes from type 2 diabetes mellitus (T2DM) patients, a finding closely associated with the onset, progression, and complications of T2DM-related keratopathy [63]. The pathological state is also reflected in in vitro models; a co-culture model of immortalized HCECs overlaid onto HCFs demonstrated that T2DM constructs exhibited epithelial defects, thicker stromal layers, and a reduction in both EV particle size and number, alongside decreased TSP-1 expression [26].

Therapeutic application of exogenous exosomes has been shown to facilitate the healing of diabetic corneal injuries. Promotion of corneal epithelial repair in diabetic mice was achieved through subconjunctival injection of BMSC-Exos, with efficacy comparable to the injection of BMSCs themselves [53]. Further investigation revealed that mADSC-EVs promote diabetic corneal epithelial wound healing by activating the DC-mediated NGF/TrkA pathway [49]. Potential therapeutic value for corneal wound healing in both T1DM and T2DM patients has also been proposed for salivary exosomes [21]. To address the inflammatory component, a targeted approach was developed using MSC-Exos as carriers for c-Rel-specific siRNA (siRel). c-Rel, a member of the NF-κB family expressed in activated immune cells, regulates multiple inflammatory factors. This exosome-based delivery system demonstrated superior efficacy in treating corneal injury in T1DM mice compared to nanopolymer-based delivery [61].

## 6. Therapeutic Effects of Exosomes on Other Ocular Surface Diseases

### 6.1. Keratoconus

Keratoconus (KC) is a degenerative, non-inflammatory corneal ectatic disorder [170] characterized by bilateral, asymmetric corneal thinning, enlargement, and increased curvature. Progressive myopia and irregular astigmatism in the early stages often lead to corneal scarring and significant vision loss. The pathogenesis is complex, involving genetic, environmental, and biomechanical factors [171], with current management relying on corneal collagen cross-linking, specialized contact lenses, or corneal transplantation [172]. Significant promise is now being shown by exosomes in both the understanding and treatment of KC.

Initial characterization of the exosomal landscape in KC was provided by the identification and isolation of tear-derived EVs from patients [94]. Compared to healthy controls, reduced levels of CD63^+^/CD9^+^ and CD63^+^/CD81^+^/CD9^+^ EVs were observed in the tears of male patients, though a higher relative total number of tear EVs was noted in males compared to females [94]. Further evidence of exosomal involvement was demonstrated by differences in the protein and miRNA expression profiles of exosomes secreted by HCKs from KC patients compared to those from healthy individuals [93]. These compositional alterations may be partly attributed to disruptions in the exosome biogenesis machinery, as alterations in components of the ESCRT were observed within KC-derived HCKs, affecting ubiquitin-mediated cargo selection [173]. Comprehensive characterization of exosomes from KC and healthy HCKs has identified multiple pathologically relevant miRNAs, proteins, and gene expression abnormalities, suggesting that exosomes from healthy sources may possess therapeutic potential for counteracting these disease-driving signals [95]. Exploration of alternative exosome sources has revealed that high concentrations of salivary exosomes can significantly upregulate cleaved wavefront protein and TSP-1 while downregulating fibronectin in KC-derived HCKs in vitro, indicating a potential reparative effect [21]. Collectively, this research enhances the understanding of KC pathophysiology and positions exosomes as promising diagnostic markers and future therapeutic tools.

### 6.2. Keratitis

#### 6.2.1. Fungal Keratitis

Fungal keratitis (FK) is a severe infectious disease that compromises ocular surface defenses, leading to corneal inflammation, ulceration, and potential permanent visual impairment or loss of the eye. *Candida albicans* and *Aspergillus fumigatus* (AF) are common pathogens, with the prevalence of *Candida* keratitis rising due to antibiotic/corticosteroid abuse and contact lens wear [174]. The prognosis is often poor, hampered by delayed diagnosis and limited drug penetration. A novel therapeutic approach involves the use of fungal exosomes to modulate the local immune response for improved fungal clearance and prognosis.

Modulation of immune cell function and exertion of protective effects in *Candida* keratitis has been demonstrated for *Candida albicans*-derived EVs in in vivo and in vitro models [87]. Similarly, promotion of inflammatory cytokine expression in immune cells and a reduction in *Sporothrix* survival in vitro were observed with *Aspergillus fumigatus*-derived EVs (AF-EVs) [91]. Subconjunctival injection of AF-EVs in mice was also shown to increase secretory IgA (sIgA) in tears and mitigate the severity of keratitis [91]. From a pathogenesis perspective, transfer of let-7b-5p from *Aspergillus fumigatus*-treated HCECs to macrophages via exosomes has been revealed, a process that suppresses SOCS-1 expression and promotes pro-inflammatory M1 macrophage activation, a critical step in the innate immune response against FK [92].

#### 6.2.2. Bacterial Keratitis

*Pseudomonas aeruginosa* (PA), a Gram-negative opportunistic pathogen, is the most common cause of infectious keratitis, often exploiting corneal trauma or contact lens wear to initiate severe infection and corneal liquefaction [175]. A critical role for host-derived EVs in the inflammatory response has been established; EVs released from PA-infected CECs were found to promote pro-inflammatory cytokine production in naive epithelial cells and independently mediate neutrophil chemotaxis [85]. Distinct effects on CECs and neutrophils were confirmed through proteomic analysis comparing PA-derived EVs with free proteins, underscoring the role of EVs in disrupting innate immunity [84]. Furthermore, a significant upregulation of the metabolite palmitoyl-carnitine (PAMC) was identified in EVs from PA-infected cells, which induces IL-8 secretion and neutrophil migration [86]. A potential host defense mechanism was also suggested by the finding that PA can be eradicated within infected cells through the release of PAMC-laden EVs [86].

#### 6.2.3. Viral Keratitis

Herpes simplex keratitis (HSK), primarily caused by herpes simplex virus type 1 (HSV-1), is a leading cause of infectious blindness due to its recurrent nature, which can lead to corneal scarring and neovascularization [176]. A crucial role in maintaining corneal epithelial integrity and protecting against viral invasion is played by tear-derived exosomes, making them potential therapeutic targets. Metabolomic analysis of tear EVs from HSK patients via LC-MS/MS has confirmed a close association between their metabolites and disease pathophysiology, providing insights for liquid biopsy development and target identification [90]. Furthermore, a mechanism for viral persistence and transmission has been suggested by the discovery that HSV-1 genes can be transferred between cells via tear exosomes, which may serve as latent reservoirs for the virus in recurrent HSK [88].

#### 6.2.4. Acanthamoeba Keratitis

Acanthamoeba keratitis (AK) is a rare but devastating corneal infection. The protein composition of *A. castellanii* exosomes has been identified via LC-MS/MS analysis, revealing a predominance of hydrolases and oxidoreductases [89]. Experimental validation confirmed that these EVs possess aminopeptidase activity and can induce an immune response in human monocytes/macrophages, leading to target cell destruction, thus providing novel therapeutic insights [89]. Furthermore, protein components associated with pathogenesis, particularly those involved in adhesion, have been identified in *Acanthamoeba* T5 EVs using polyclonal antibodies, offering an important tool for diagnosing and treating these infections [177]. Further research is required to fully elucidate the specific role of *Acanthamoeba* EVs in disease pathogenesis.

### 6.3. Allergic Conjunctivitis

Allergic conjunctivitis (AC) is an inflammatory condition of the conjunctival epithelium, characterized by symptoms including eyelid itching, tearing, conjunctival redness, foreign body sensation, mucous discharge, and eyelid swelling [178]. Ocular surface stability is maintained by mucin secretion from CGCs, a process that is disrupted during AC. Inflammation can lead to either insufficient or excessive mucin secretion, resulting in ocular surface instability and visual impairment. The onset of AC is marked by the rapid degranulation of sensitized mast cells and the release of large amounts of histamine, which promotes the secretion of pro-inflammatory mediators and amplifies the inflammatory cascade. Binding of histamine to receptors on CGCs directly induces excessive mucin secretion [179]. To counteract this, the sequential appearance of specialized pro-resolving mediators (SPMs) at the inflammatory site serves to alleviate symptoms. A significant increase in SPM secretion via EVs was observed in female human CGCs 18 h after histamine stimulation [14]. This SPM level in female EVs was further increased by the addition of docosahexaenoic acid (DHA), an effect not observed in male cells, providing novel insights into the influence of gender differences on AC susceptibility and potential treatment strategies [14].

### 6.4. Pterygium

Pterygium is an OSD characterized by fibrovascular proliferation, wherein conjunctival tissue progressively invades the cornea. Symptoms such as eye fatigue, irritation, dryness, tearing, and a foreign body sensation are common, with potential vision loss resulting from obstruction of the visual axis, induced astigmatism, or tear film disruption [180]. The etiology is multifactorial, associated with UV exposure, viral infections, genetic factors, growth factors, and oxidative stress [181], which ultimately lead to the centripetal proliferation of altered limbal cells and disruption of the corneal–conjunctival barrier. Alterations in the ocular surface microbiome have been implicated; reduced microbial diversity and abundance, including decreased counts of *Bacillus coagulans* (BC), were identified in the tear samples of pterygium patients via 16S rRNA sequencing [181]. Internalization of BC-EVs by conjunctival epithelial cells was demonstrated, with these EVs promoting cell proliferation via the p53/CDKN1A signaling pathway while suppressing TGF-β-induced epithelial damage, thereby influencing pterygium progression [182]. Furthermore, significantly upregulated expression of fibrosis and inflammatory signaling markers was found in the stroma of pterygium tissue compared to normal conjunctival stroma [104]. In an experimental model, exosomes derived from M1 macrophages that had been pretreated with EGF were shown to suppress the inflammatory signaling, angiogenesis activation, and excessive proliferation in pterygium fibroblasts that are normally induced by untreated M1 macrophage-derived exosomes [104].

### 6.5. Transplant Rejection

Corneal transplantation is a vital surgical intervention for end-stage corneal diseases. However, in high-risk patients with pre-existing corneal neovascularization and compromised immune privilege, graft failure rates can reach 70%, primarily due to immune rejection [183]. The role of exosomes in this process is complex. In a mouse MHC-mismatched (C57BL/6-to-BALB/c) corneal transplantation model, significant enrichment of the donor-derived MHC class I molecule H2-Q2 was observed in the serum EVs of recipients with rejected grafts. This suggests that donor-derived vesicles may contribute to rejection by facilitating antigen cross-presentation and host T cell activation. In contrast, high expression of serum amyloid A2 was associated with the non-rejected group, potentially promoting immune tolerance through macrophage phenotype regulation [116]. From a therapeutic perspective, the beneficial effects of exosomes have been demonstrated. In an allogeneic corneal transplant rejection model in Wistar-Lewis rats, subconjunctival injection of MSC-Exos was shown to effectively prolong corneal graft survival. The mechanism is believed to involve the ability of MSC-Exos to traverse biological barriers and directly act on target tissues, where they significantly suppress the pro-rejection Th1 immune response [115].

## 7. Recent Advances in Optimizing Exosome-Based Drug Delivery Systems

As natural nanoscale carriers, exosomes share key characteristics with cell membranes, including their small size and negative surface charge. Compared to synthetic delivery systems, they offer distinct advantages such as low immunogenicity, minimal toxicity, high stability, inherent targeting capabilities, and an innate ability to cross biological barriers. These properties collectively contribute to reduced systemic side effects, enhanced bioavailability, and improved therapeutic efficacy, establishing exosomes as a highly promising platform for drug delivery. However, limitations are associated with natural exosomes, including inherent disease non-specificity and limited retention on the ocular surface. Consequently, significant research efforts have been directed toward optimizing exosome-based delivery through combination with other components. Strategies include achieving synergistic therapeutic effects by conjugating exosomes with drugs, nucleic acids, or gene therapy agents, as well as enhancing delivery stability and persistence through formulation with bioadhesive materials. Concurrently, more precise conjugation techniques are under continuous development. Furthermore, practical challenges related to storage must be considered, as traditional long-term preservation requires rapid cryopreservation in liquid nitrogen, followed by transfer to −80 °C or liquid nitrogen tanks, processes that incur high costs and logistical complexity.

Exosomes serve as an excellent platform for gene delivery applications, facilitating the transport of diverse payloads including miRNAs, siRNAs, therapeutic proteins, and CRISPR-based gene-editing tools. Substantial progress has been made in the field of nucleic acid delivery, where conventional loading methods such as electroporation, sonication, and freeze–thaw cycles often compromise vesicle integrity and exhibit low encapsulation efficiency [76]. An innovative approach was pioneered using DNA zipper-mediated membrane fusion to fuse siRNA-loaded liposomes with CEC-Exos, creating hybrid exosomal vesicles for tissue-specific siRNA delivery [76]. This method demonstrated effective targeting of corneal cells and successful intracellular siRNA delivery, yielding favorable therapeutic outcomes in a DED mouse model. A similar strategy was employed to combine c-Rel-specific siRNA with exosomes for topical application, effectively accelerating wound healing in both normal and diabetic corneas, with efficacy surpassing that of siRNA-loaded nanopolymers [61]. Furthermore, successful suppression of corneal inflammatory fibrosis and promotion of epithelial migration have been achieved through exosomal delivery of miR-29b-3p and miR-24-3p, respectively [31,42]. In the context of therapeutic protein delivery, Yu et al. [60] utilized an MMP-cleavable peptide chain to conjugate an anti-tumor necrosis factor-α antibody (aT) onto the surface of ADSC-Exos, generating engineered aT-Exos with synergistic effects. Compared to aT alone, unmodified exosomes, or a simple mixture of aT and exosomes, aT-Exos demonstrated superior efficacy in alleviating corneal injury. Exosome-mediated delivery of CRISPR/Cas9 represents a paradigm shift in gene therapy, offering hope for curative treatment of hereditary ocular diseases. Ocular conditions caused by single-gene mutations, such as Fuchs’ endothelial corneal dystrophy and corneal stromal dystrophies, hold promise for gene correction. For infectious diseases like herpes simplex keratitis, CRISPR systems could directly target and eliminate latent viral genomes, achieving a “curative” therapeutic outcome. Although studies have demonstrated the feasibility of exosome-mediated CRISPR delivery in diseases such as liver disorders and cancer [184,185], this application in the ocular surface field remains in its infancy, with tremendous potential yet to be explored.

In the realm of synergistic antioxidant and anti-inflammatory therapy, a novel therapeutic nanoparticle was developed through the in situ growth of cerium oxide nanocrystals on the surface of MSC-derived exosomes [73]. This construct exhibits excellent biocompatibility and synergistically enhances ROS scavenging and anti-inflammatory capacity, alleviating symptoms of DED and even reversing pathological alterations at cellular and tissue levels. A similar effect was achieved through the in situ deposition of ascorbic acid-reduced gold nanoparticles onto the phospholipid membranes of MSC-derived exosomes [69].

For drug and biologic delivery, targeted delivery of dexamethasone to inflammatory cells was accomplished by loading it into bovine milk exosomes using ultrasound [50]. This approach enhanced therapeutic efficacy by prolonging hormone activity and reducing administration frequency without significant side effects. Superior efficacy in alleviating corneal injury was demonstrated by these engineered exosomes compared to antibody alone, unmodified exosomes, or a simple mixture of both components. An innovative approach to addressing hypoxia involved the creation of OExo-NPs through the ultrasonic treatment of a mixture of ADSC-Exos and hemoglobin solution [25]. Application of OExo-NPs in corneal epithelial cell scratch assays effectively alleviated hypoxia while inhibiting angiogenesis, inflammation, and scar formation. Finally, a composite was formed by combining PEVs with the anti-angiogenic agent kaempferol (PEV-KM) [102]. This compound was efficiently internalized by human vascular endothelial cells, downregulated angiogenesis-related genes, and significantly inhibited neovascularization in an alkali-induced corneal neovascularization mouse model, while concurrently reducing pro-angiogenic and inflammatory cytokines.

The clinical translation of exosome-based therapies for OSDs remains constrained by challenges related to cost-effectiveness and bioavailability. Topical administration, while being the most common and desirable route, is significantly limited by rapid clearance mechanisms such as blinking, the tear film, and the tight junction barriers of the corneal epithelium. To address these limitations, innovative formulation strategies are being actively developed.

Microneedle technology has recently been adapted for ocular use as a minimally invasive delivery method. Incorporation of polyvinyl alcohol-soluble microneedles into an exosome solution was shown to produce eye drops with excellent biocompatibility that significantly increased exosomal retention time on the ocular surface and enhanced corneal permeability [60].

Hydrogel-based systems have emerged as particularly promising platforms due to their hydrophilic, cross-linked polymer networks. Advantages such as tunable mechanical properties, high biocompatibility, and favorable optical characteristics have enabled their application for exosome delivery to ocular tissues [186]. A thermosensitive hydrogel (THH) was developed by modifying HA with dimethylethylene glycol dimethacrylate (DEGMA) for the controlled release of miR-24-3p-rich exosomes [42]. This formulation created a uniform, clear layer on the ocular surface that resisted clearance by blinking, thereby enhancing stability and prolonging contact time. In a different approach, gelatin methacrylate (GelMA) was synthesized and used as a scaffold to culture MSCs, yielding three-dimensional exosomes (3D-Exos) with higher production yields and superior therapeutic properties [55]. Subsequent encapsulation of these 3D-Exos within a GelMA hydrogel created a multifunctional sustained-release system that restored corneal morphology and function more effectively than systems using conventional two-dimensional exosomes. Furthermore, a hydrogel constructed through dynamically reversible Schiff base reactions between oxidized guar gum and carboxymethyl chitosan demonstrated potent tissue adhesion at physiological temperatures [51]. Incorporation of MSC-derived exosomes into this adhesive hydrogel significantly improved wound repair in a rabbit corneal defect model by enhancing collagen deposition and reducing inflammation.

Methylcellulose formulations have also been utilized as biocompatible adhesives and lubricants to enhance EV bioavailability. Embedding of EVs within methylcellulose was demonstrated to effectively prolong their in vivo duration of action, thereby reducing the required administration frequency [38].

Beyond delivery systems, stabilization technologies are critical for practical clinical application. Lyophilization is a widely used technique for enhancing the stability of nanoscale drug delivery systems. The natural protective molecule ectoine, which enables bacterial survival under extreme conditions by forming a stable hydration layer, has been investigated for this purpose. A systematic evaluation of ectoine’s cryoprotective effect on milk-derived EVs during lyophilization was conducted, demonstrating that the addition of trehalose and 0.5–4% ectoine allowed key biological properties to be retained for over two months [72]. These lyophilized milk-EVs effectively protected HCECs from hypertonic-induced damage and demonstrated superior therapeutic efficacy in a benzalkonium chloride-induced DED rabbit model. This approach addresses critical pharmaceutical requirements for stable exosome formulations, eliminates the reliance on −80 °C storage, and establishes lyophilization as a transformative preservation method for exosome-based therapies.

Despite the promising advances in engineered exosome applications—including endogenous and exogenous modifications, the development of novel multi-functional drug delivery platforms, and improved storage stability—we must consider the scalability and manufacturing feasibility of translating these innovations into routine ophthalmic practice. Direct loading of therapeutic agents onto or into exosomes via physical or chemical methods offers higher efficiency and demonstrates certain feasibility. However, this process may compromise membrane integrity or leave chemical residues, necessitating further development of more refined techniques suitable for clinical application. Genetic engineering of donor cells to modify exosomal protein or nucleic acid content offers lower production costs and improved batch-to-batch consistency, yet suffers from limited specificity and efficiency [187]. Exosome loading efficiency and drug release kinetics are influenced by multiple factors, including drug type, loading method, and exosome source. Their pharmacokinetic profiles remain incompletely understood, underscoring the urgent need for more clinical trials to evaluate the safety and efficacy of exosome-based drug formulations. Furthermore, the lack of stability studies and standardized technical protocols presents challenges for regulatory approval and industrial-scale production.

## 8. Clinical Translation Challenges and Future Directions

### 8.1. Current Status of Clinical Translation of Exosomes in the Ocular Surface Field

Clinical translation of therapeutics requires a stepwise process: from laboratory target identification and drug screening optimization, to preclinical studies evaluating efficacy and safety in animal models, to clinical trials assessing safety and efficacy in humans, and finally to post-marketing long-term monitoring of therapeutic effects and adverse reactions. The application of exosomes in the ocular surface field remains largely at the preclinical stage. Their therapeutic mechanisms and effects have been extensively validated in cell culture systems, animal models, and ex vivo experimental models. Exosomes carry bioactive molecules such as proteins and miRNAs, exerting therapeutic effects through mechanisms including immune regulation, inflammation suppression, and promotion of cell proliferation, demonstrating significant potential in various ocular surface diseases including DED and corneal injury.

Several studies have advanced to the clinical trial stage. Zhou et al. [81] conducted a clinical trial enrolling 14 patients with refractory GVHD-associated DED, administering eye drops containing hUCMSC-Exos four times daily in each eye for two weeks. Patients exhibited significant symptom relief, with no MSC-Exo-related complications reported. Habibi et al. [188] conducted the world’s first triple-blind randomized controlled trial for SS-associated DED, enrolling eight participants. The treated eyes received 10 µg of hUCMSC-Exos eye drops twice daily for two weeks, with three months of follow-up. Results demonstrated significant symptomatic improvement in the treatment group compared to controls.

However, existing clinical trials are limited by small sample sizes and short follow-up periods, restricting the assessment of treatment generalizability and long-term safety. Notably, Habibi et al. reported that the therapeutic effects of short-term exosome administration were limited in duration, with a majority of underlying factors deteriorating three months post-treatment. These findings underscore the need to evaluate the potential benefits of long-term, sustained exosome therapy, as well as possible adverse effects similar to those associated with corticosteroids or immunosuppressive agents, such as cataract formation, glaucoma, or infection risk.

### 8.2. Key Barriers to Clinical Translation

#### 8.2.1. Scalability Challenges in Production

The therapeutic dose of exosomes required for clinical applications can reach up to 10^11^ particles per patient a demand far exceeding the output of conventional culture methods [11]. Scalable production faces several core challenges. Regarding cell sources, primary mesenchymal stem cells exhibit donor-dependent variability and undergo senescence during in vitro expansion, compromising exosome quality and yield. In terms of culture systems, while three-dimensional platforms can enhance productivity, they introduce issues such as shear stress damage, difficulties in cell harvesting, and the need for cell-type-specific optimization of process parameters. Downstream purification presents another bottleneck: no single method achieves high yield, purity, and bioactivity simultaneously, while combining multiple techniques increases process complexity and cost.

#### 8.2.2. Quality Control and Standardization

Quality control for exosome-based therapeutics encounters three major hurdles. First, the lack of definitive markers to distinguish exosomes from other extracellular vesicles (e.g., microvesicles) creates ambiguity in product classification and regulatory pathways. Second, current characterization techniques (NTA, TEM, Western blot) are inadequate for fully capturing vesicle heterogeneity; function depends on the integrated structure rather than individual components, and potency assays remain underdeveloped. Third, although the MISEV2023 guidelines have been published, adherence to these updated standards has not yet become routine practice. Existing frameworks for cell and gene therapy products do not fully address the unique characteristics of exosomes [121], creating challenges for both product development and regulatory review.

#### 8.2.3. Unknown Long-Term Safety and Mechanistic Ambiguity

Immunogenicity risks vary with exosome source and modification strategies. Bacterial outer membrane vesicles containing LPS may trigger inflammation, tumor-derived exosomes carry potential pro-oncogenic risks [189], and engineered modifications may alter immune profiles and provoke unintended responses [187]. Data on long-term immunotoxicity, tumorigenicity, and reproductive toxicity remain scarce. Mechanistically, native exosomes contain hundreds of bioactive molecules, making it difficult to determine whether therapeutic effects are driven by a single component or synergistic pathways. This “mechanism gap” directly impedes the establishment of robust quality control standards. Furthermore, the in vivo fate of exosomes—including biodistribution, metabolism, and clearance—remains poorly understood, as current tracing technologies lack the precision and duration needed for quantitative long-term tracking.

#### 8.2.4. Duration of Therapeutic Effect and Dosing Regimens

In animal studies, the duration of exosome therapeutic effects varies considerably depending on the disease model, administration route, and dosing frequency. In DED models, common dosing regimens include topical instillation 2 to 4 times daily for 1 to 2 weeks, with follow-up periods typically ranging from 1 to 2 weeks. One study extended the follow-up period to 8 weeks, suggesting that therapeutic effects may persist to some extent after treatment cessation [64]. In SS-associated DED models, treatment is often administered via intravenous or subconjunctival injection, with follow-up periods ranging from 4 to 8 weeks. Several studies have demonstrated that improvements in immune modulation and glandular function can persist through the end of follow-up [110,112,114]. In corneal injury models, following single or multiple topical administrations, follow-up periods generally range from 1 day to 2 weeks, with therapeutic effects sustained throughout this period. Some studies designed longer follow-up periods, such as 8 weeks [51], indicating that exosome therapy may offer a degree of durability.

However, the majority of current studies lack systematic evaluation of whether therapeutic effects persist after treatment cessation. Most experimental designs assess efficacy immediately or shortly after treatment completion, failing to clearly distinguish between “short-term improvement” and “long-term structural or functional recovery.” Furthermore, whether repeated administration is required to maintain efficacy remains to be systematically investigated. Overall, existing animal studies demonstrate that exosome therapy exerts clear short-term efficacy, but its long-term translational potential requires further validation through extended follow-up periods, assessment of functional recovery endpoints, and evaluation of maintenance dosing regimens. Future studies should explicitly address the relationship between duration of therapeutic effect, dosing frequency, and follow-up duration at the experimental design stage to enhance clinical translational value. Dedicated pharmacokinetic/pharmacodynamic studies utilizing advanced tracing technologies are urgently needed to inform rational dose selection and dosing frequency.

#### 8.2.5. Challenges in Exosome Engineering

A fundamental tension exists between loading efficiency and membrane integrity. Endogenous loading approaches yield low efficiency (5–30%) and offer limited control over cargo packaging [190]. Exogenous methods (electroporation, sonication, extrusion, saponin treatment) achieve higher loading but often compromise membrane structure, induce protein denaturation, or cause siRNA aggregation, all of which can alter in vivo behavior. Surface modification with targeting peptides may introduce immunogenicity and elicit anti-drug antibodies, undermining therapeutic efficacy. Quality control for engineered exosomes is further complicated by the need to assess additional parameters such as encapsulation efficiency, drug loading capacity, and positivity rate—metrics for which standardized assays and reference materials are largely unavailable.

### 8.3. Future Directions and Perspectives

In the therapeutic landscape of ocular surface diseases, exosomes are positioned as a complementary rather than replacement strategy. Their distinct advantages—multitarget mechanisms, intrinsic immunomodulatory properties, natural tropism for corneal tissues, and favorable safety profile—make them particularly valuable in specific contexts: complex multifactorial conditions such as moderate-to-severe DED, diseases requiring tissue regeneration including corneal injury and limbal stem cell deficiency, patient refractory to conventional therapies, and as engineered platforms for delivering sensitive biologics [187]. However, exosomes are unlikely to supplant established treatments for acute infectious keratitis, mild -DED, or cost-sensitive large-scale applications, where conventional small molecules and artificial tears will remain the mainstream choice. Future exosome research should focus on building intelligent, standardized, and scalable systems for development and manufacturing. At the mechanistic level, single-vesicle analysis technologies, multi-omics approaches, and AI-driven data mining will be essential for dissecting exosome heterogeneity and its relationship to biological function, elucidating the mechanisms of different exosome subpopulations. The use of alternative models such as 3D cell culture and organoid technologies to simulate the human tissue environment represents a promising new direction [191]. At the engineering level, there is a need to develop gentle, efficient, and controllable drug loading techniques (e.g., click chemistry, microfluidic electroporation) and programmable targeting platforms (e.g., synthetic biology-derived “smart” exosomes) for precise regulation of vesicle structure and function [190]. At the manufacturing level, establishing GMP-compliant closed-system automated production lines, integrating Quality by Design principles into process control, and promoting the issuance of specific regulatory guidelines for exosome-based drugs are urgent priorities [192]. At the clinical application level, systematic evaluation of pharmacokinetics, biodistribution, and long-term safety should be conducted based on disease characteristics, along with exploration of combination therapies and personalized treatment strategies. With the deepening integration of interdisciplinary technologies and the parallel advancement of regulatory science, exosome-based therapeutics are poised to overcome current bottlenecks, transitioning from laboratory research to clinical practice and emerging as important tools in the era of precision medicine.

### 8.4. Current Limitations of Exosome-Based Therapies for Ocular Surface Diseases

Despite the promising preclinical results summarized in this review, several limitations must be acknowledged before exosome-based therapies can advance to clinical application. Lack of long-term safety data: Most preclinical studies have evaluated exosome treatment over relatively short periods (days to weeks). Systematic assessments of chronic toxicity, immunogenicity, tumorigenic potential, and reproductive toxicity following repeated administration remain absent. Due to limitations in current tracing technologies, the long-term fate of exosomes in ocular tissues—including accumulation, metabolism, and clearance—remains poorly understood. Absence of large-animal translational models: The majority of in vivo evidence derives from small-animal models (mice, rats), which do not fully recapitulate the anatomy, immune microenvironment, or wound healing dynamics of the human ocular surface. Large-animal models (rabbits, pigs, non-human primates) that better approximate human ocular physiology are urgently needed to validate efficacy, optimize dosing regimens, and assess safety prior to first-in-human trials. No completed randomized controlled human clinical trials. To date, most therapeutic evidence for exosome-based interventions in ocular surface diseases remains preclinical. Published Phase I/II/III trials are extremely limited, and the safety, tolerability, and efficacy in patients have not yet been established. Uncertainty regarding optimal dose, administration route, and treatment frequency. Preclinical studies have employed highly variable dosing regimens—ranging from single doses to daily or weekly repeated administration—with no consensus on the minimum effective dose, optimal dosing interval, or most appropriate route (topical eye drops, subconjunctival injection, hydrogel-based delivery) for different disease indications. Unknown durability of therapeutic effects: Whether observed benefits—such as corneal re-epithelialization, reduced fibrosis, or improved tear secretion—translate into sustained functional recovery remains unclear. Most studies report outcomes within days to weeks, and long-term follow-up data on disease recurrence, visual function, and quality of life are lacking.

## 9. Conclusions

This review systematically highlights the considerable potential of exosomes, as a versatile cell-free therapeutic platform and drug delivery vehicle for a wide spectrum of OSDs. Substantial evidence demonstrates that exosomes derived from diverse sources—including MSCs, epithelial cells, and other somatic cells—can effectively modulate key pathological processes through their rich cargo of bioactive molecules. These processes include immune-inflammatory responses, oxidative stress, cell proliferation and migration, and tissue remodeling. Promising therapeutic efficacy has been consistently reported across multiple preclinical models of OSDs, such as DED, corneal injury, keratoconus, infectious keratitis, allergic conjunctivitis, pterygium, and corneal transplant rejection. Furthermore, the therapeutic profile of natural exosomes can be significantly optimized through various engineering strategies. Surface modifications, specific cargo loading, and combination with biocompatible materials like hydrogels have been shown to enhance targeting precision, improve ocular retention, and prolong therapeutic action, thereby paving new avenues for precise, efficient, and sustained treatment of ocular surface pathologies. Although challenges in standardization, scalable production, and clinical validation remain, the majority of therapeutic evidence remains at the preclinical and early clinical stage. The continued deepening of our understanding of exosome biology, coupled with relentless innovation in bioengineering technologies, positions exosome-based therapies as a transformative modality poised to reshape the therapeutic landscape for OSDs in the near future.

## Figures and Tables

**Table 1:** Summary of preclinical studies on exosome-based therapies for ocular surface diseases.

Author & Year	Cell Type/Tissue-Origin of Exosomes *	Exosome Isolation #	Content Analyzed	Exosome Analysis Methods ^	Disease Model	Level of Evidence	Main Finding	Animal Species	Administration Route	Administered Dose	Dosing Frequency	Treatment Duration	Follow-Up Period	Duration of Therapeutic Effect
(Lee and Dartt et al., 2024) [14]	HCjGC	PEG	Lipid mediators	TEM, NTA, WB	Allergic conjunctivitis	In vitro study	female CjGCs increase SPMs							
(Buono et al., 2021) [15]	BMSC, serum	DUC	miRNA	FC, NTA, WB	Corneal endothelial dystrophy	In vitro study	protect against ER stress							
(Altug et al., 2024) [16]	CSSC	DUC	miRNA, RNA	FC, TEM, DLS	Corneal injury	In vitro study	antifibrotic and promote regeneration							
(An et al., 2023) [17]	BMSC	DUC	None	NTA	Corneal injury	In vitro study	mediate wound healing							
(Bonelli et al., 2025) [18]	BMSC	SEC	None	NTA, TEM, WB, FC	Corneal injury	Observational study Ex vivo study	enhance epithelial repair							
(Desjardins et al., 2022) [19]	HCEC, HCF, HCEnC	DUC	None	NTA, TEM, WB, DLS	Corneal injury	In vitro study	promote wound healing							
(Donohoe et al., 2025) [20]	MSC	DUC, SEC	None	NTA, TEM, FC	Corneal injury	Animal model In vitro study	TGF-β1-licensed MSC-sEV reduce inflammation	mouse	topical/subconjunctival injection	Topical: 10 μL Subconjunctival: 30 μL	Topical: days 0, 1, 3 Subconjunctival: days 0, 3	3 days	2 weeks	2 weeks
(Escandon et al., 2022) [21]	Salivary	None	None	ExoView	Corneal injury	In vitro study	regulate wound healing							
(Han et al., 2015) [22]	mCF	DGUC	Protein	SDS-PAGE	Corneal injury	In vitro study	MMP14-containing in angiogenesis							
(Han et al., 2017) [23]	CEC	DUC, PEG	Protein	EM, DLS, WB, LC-MS/MS	Corneal injury	In vitro study	mediate corneal communication							
(Han et al., 2019) [24]	mCF	DUC, PEG	Protein	TEM, NTA, WB	Corneal injury	In vitro study	MMP14-containing cleave VEGFR1							
(Han et al., 2025) [25]	ADSC	DUC	None	NTA, TEM, SEM, DLS	Corneal injury	In vitro study	OExo-NPs alleviate hypoxia							
(Hefley et al., 2024) [26]	HCEC, HCF	DUC	Protein	ExoView	Corneal injury	Observational study Ex vivo study	diabetes alters composition							
(Hu et al., 2022) [27]	HAEC	DUC	miRNA, protein	NTA, TEM, WB	Corneal injury	Animal model In vitro study	promote ECM reorganization	rabbit	topical/subconjunctival injection	Topical: 40 μL Subconjunctival: 100 μL	Topical: 3 times daily Subconjunctival: twice weekly	2 weeks	2 weeks	2 weeks
(Lee et al., 2024) [28]	iPSC	PEG	miRNA	NTA, TEM, WB	Corneal injury	Animal model	hiPSC-RO enhance wound healing	mouse	topical instillation	5 μL	3 times (0, 10, 20 min post-injury)	/	36 h	36 h
(Lee et al., 2025) [29]	M1 macrophage, M2a macrophage	PEG	None	NTA	Corneal injury	In vitro study	M2a macrophage microenvironment promotes HCEC healing							
(Liang et al., 2025) [30]	Salivary	None	None	None	Corneal injury	Animal model In vitro study	promote wound healing	mouse	topical instillation	10 μg	twice daily	3 days	3 days	3 days
(Liu et al., 2024) [31]	BMSC	DUC	miRNA	TEM, NTA, WB	Corneal injury	Animal model In vitro study	miR-29b-3p activates autophagy	mouse	topical/subconjunctival injection	5 μL	Topical: 3 times daily (days 1–7) Subconjunctival: twice weekly (days 8–14)	2 weeks	2 weeks	2 weeks
(Ma et al., 2025) [32]	ADSC	DUC	None	TEM, NTA	Corneal injury	Animal model In vitro study	inhibit apoptosis and scarring	rat	topical instillation	7.5 μL	single dose	/	2 weeks	2 weeks
(Mckay et al., 2020) [33]	HCEC	DUC	Protein	TEM, WB, STED, IF, MS	Corneal injury	In vitro study	promote myofibroblast differentiation							
(Meissner et al., 2024) [34]	ADSC	DUC	None	FC	Corneal injury	In vitro study	mitigate ER stress							
(Nuzzi et al., 2021) [35]	BMSC	DUC	None	NTA	Corneal injury	In vitro study	promote HCEC regeneration							
(Ong et al., 2023) [36]	ESC-MSC	TFF	None	NTA, WB, ELISA	Corneal injury	Animal model In vitro study	reduce corneal scarring	rat	topical instillation	8 μL	6 times daily	5 days	5 days	5 days
(Ryu et al., 2023) [37]	ADSC	SEC	miRNA	IF, NGS	Corneal injury	In vitro study	promote CEC regeneration							
(Saccu et al., 2022) [38]	BMSC	DUC	None	NTA, FC, TEM, WB, ExoView	Corneal injury	Animal model In vitro study	regulate inflammation and angiogenesis	mouse	topical instillation	10 μL	twice daily for 5 days, then every other day	2 weeks	2 weeks	2 weeks
(Samaeekia et al., 2018) [39]	CSSC	DUC	None	TEM, DLS, WB	Corneal injury	Animal model In vitro study	accelerate wound healing	mouse	topical instillation	5 μL	4 times (0, 10, 20, 30 min post-injury)	/	1 day	1 day
(Saraf et al., 2024) [40]	Serum	SEC	Metabolite, protein	NTA, TEM, WB, LC-MS/MS, ELISA	Corneal injury	In vitro study	retain wound healing without inflammation							
(Shojaati et al., 2019) [41]	CSSC	PEG, DUC	miRNA	TRPS, WB, TEM, FC, miRNA-Seq	Corneal injury	Animal model In vitro study	deliver anti-fibrotic miRNAs	mouse	topical administration	10^9^ particles/mL (in 1 μL fibrin gel)	single dose	/	4 weeks	4 weeks
(Sun et al., 2023) [42]	ADSC	DGUC	miRNA	TEM, FC, WB, NTA	Corneal injury	Animal model In vitro study	miRNA 24-3p-rich promote epithelial healing	rabbit	topical/subconjunctival injection	/	Decreasing frequency	4 weeks	4 weeks	4 weeks
(Tao et al., 2019) [43]	hP-MSC	DUC	None	ELISA, TEM, DLS	Corneal injury	Animal model	promote corneal wound healing	mouse	topical instillation	10 μL	3 times daily	2 weeks	2 weeks	2 weeks
(Tati et al., 2024) [44]	BMSC	PEG	None	TEM, SEM, NTA, WB, IF	Corneal injury	In vitro study	suppress apoptosis							
(Tati et al., 2024) [45]	BMSC, HCEC	PEG	None	TEM, SEM, NTA, WB, IF	Corneal injury	In vitro study	outperform HCEC-EVs							
(Verma et al., 2023) [46]	LESC	DUC	miRNA, protein	NTA, FC, WB, IF, NGS, LC-MS	Corneal injury	In vitro study	diabetic LEC-derived alter LSC function							
(Villatoro et al., 2020) [47]	LESC	DUC	Protein	TEM, DLS, WB	Corneal injury	In vitro study	cLSC secretome inhibits fibroblast proliferation							
(Wang et al., 2020) [48]	iPSC, hUC-MSC	DUC	None	TEM, NTA, WB	Corneal injury	Animal model In vitro study	iPSC-derived outperform MSC-derived	rat	topical instillation	5 μL	4 times daily	2 days	2 days	2 days
(Wang et al., 2023) [49]	ADSC	DUC	None	NTA, TEM, WB	Corneal injury	Animal model In vitro study	activate NGF/TrkA pathway	mouse	topical instillation	10 μL	3 times daily	2 weeks	2 weeks	2 weeks
(Wang et al., 2024) [50]	Milk	DUC	Loaded drug	TEM, FC, WB	Corneal injury	Animal model In vitro study	DXMS@EXO modulates Wnt pathway	mouse	topical instillation	5 μL	twice daily	1 week	1 week	1 week
(Wei et al., 2025) [51]	MSC	None	None	NTA, TEM, WB	Corneal injury	Animal model In vitro study	OGG/CMCS hydrogel promotes healing	rabbit	topical administration	8.75 × 10^9^ particles/mL	single dose	/	8 weeks	8 weeks
(Widyaningrum et al., 2022) [52]	Platelet	DUC	Protein	AFM, NTA, DLS, WB	Corneal injury	In vitro study	promote CEC regeneration							
(Wu et al., 2023) [53]	BMSC	None	None	None	Corneal injury	Animal model	repair diabetic cornea	mouse	Subconjunctival injection	20 μL	single dose	/	3 days	3 days
(Xu et al., 2024) [54]	mAF-MSC	DUC	mRNA, Protein	None	Corneal injury	Animal model In vitro study	deliver DNMT1	mouse	injection in the corneal endothelium	10 μL	single dose	/	10 days	10 days
(Xu et al., 2025) [55]	BMSC	DUC	None	TEM, DLS, NTA	Corneal injury	Animal model In vitro study	3D-derived deliver miR-150-5p targeting *PDCD4*	rabbit	topical administration	/	single dose	/	4 weeks	4 weeks
(Yam et al., 2023) [56]	CSSC	PEG, DUC	miRNA	TRPS, FC	Corneal injury	Animal model In vitro study	miR-29a/381 identify healing CSSCs	mouse	topical administration	/	single dose	/	10 days	10 days
(Yeung et al., 2022) [57]	HCK, HCF, HCM	DUC	Protein	WB, NTA, TEM, MS	Corneal injury	In vitro study	promote epithelial migration							
(Yeung et al., 2024) [58]	HCEC, HCK, HCF, HCM	DUC	Protein	WB, NTA, TEM, MS	Corneal injury	In vitro study	have distinct protein profiles							
(Yu et al., 2022) [59]	BMSC	DGUC	None	TEM, NTA	Corneal injury	In vitro study	cornea-on-chip validates MSC-derived							
(Yu et al., 2024) [60]	ADSC	DUC	Loaded drug	TEM, FC, IF, NTA, ELISA	Corneal injury	Animal model In vitro study	aT-Exo synergistically alleviates injury	mouse	topical instillation/topical administration/subconjunctival injection	1 μg	once every 3 days	2 weeks	2 weeks	2 weeks
(Zhao et al., 2023) [61]	MSC	PEG	siRNA	TEM	Corneal injury	Animal model	exosome-siRel accelerates wound healing	mouse	topical instillation	1.25 μg	3 times daily	2 days	2 days	2 days
(Zhou et al., 2023) [62]	BMSC	PEG	None	TEM, WB, NTA	Corneal injury	Animal model In vitro study	activate p44/42 MAPK	mouse	subconjunctival injection	100 μg	once daily	1 or 2 weeks	2 weeks	2 weeks
(Chen et al., 2021) [63]	Plasma	SEC	Protein	WB, NTA, TEM, MS	Diabetic keratopathy	Observational study Ex vivo study	FLOT2 as DK biomarker							
(Chan et al., 2025) [64]	hUC-MSC	TFF	None	NTA	Dry eye disease	Animal model	reduce inflammation	rat	topical instillation	20–30 μL	twice daily	2 weeks	8 weeks	8 weeks
(Chen et al., 2025) [65]	hUC-MSC	DUC	miRNA	IF	Dry eye disease	Animal model In vitro study	miR-146a targets SQSTM1	mouse	topical instillation	5 μL	twice daily	1 week	1 week	1 week
(Cross et al., 2023) [66]	Tear	DUC	RNA	NTA, TEM, FC, WB	Dry eye disease	Observational study Ex vivo study	DED diagnostic biomarker							
(Guo et al., 2022) [67]	hUC-MSC	DUC	None	TEM, NTA, WB	Dry eye disease	In vitro study	suppress ocular inflammation via DC-Th17 inhibition							
(Lee et al., 2024) [68]	Limosilactobacillus fermentum	DUC	None	ElISA, NTA, TEM	Dry eye disease	In vitro study	probiotic-derived reduce conjunctival inflammation							
(Ma et al., 2023) [69]	BMSC	DGUC	Loaded drug	TEM, NTA, WB	Dry eye disease	Animal model In vitro study	mExo@AA synergistically treats DED	mouse	topical instillation	5 μL	twice daily	1 week	1 week	1 week
(Pucker et al., 2022) [70]	Tear film	PEG	miRNA	ELISA, TEM, RNA-Seq	Dry eye disease	Observational study Ex vivo study	contain DED-associated miRNAs							
(Ren et al., 2024) [71]	PDLSC	DUC	None	TEM, NTA, WB	Dry eye disease	In vitro study	protect goblet cells							
(Ren et al., 2025) [72]	Milk	DUC	None	TEM, NTA, DLS, WB	Dry eye disease	Animal model In vitro study	lyophilized retain therapeutic efficacy	rabbit	topical instillation	50 μL	twice daily	12 days	12 days	12 days
(Tian et al., 2023) [73]	BMSC	DUC	Loaded drug	TEM, EDS, NTA, ICP-MS	Dry eye disease	Animal model In vitro study	MSCExo-Ce scavenges ROS	mouse	topical instillation	10 μL	twice daily	1 week	1 week	1 week
(Wang et al., 2022) [74]	ADSC	DUC	None	TEM, NTA, WB	Dry eye disease	Animal model	inhibit NLRP3	mouse	topical instillation	5 μL	3 times daily	1 week	1 week	1 week
(Wang et al., 2023) [75]	hUC-MSC	DUC	miRNA	TEM, NTA, WB, miRNA-Seq	Dry eye disease	Animal model	target IRAK1/TAB2/NF-κB	mouse	topical instillation	5 μL	4 times daily	3 weeks	3 weeks	3 weeks
(Xie et al., 2024) [76]	CEC	DUC	siRNA	DLS, NTA, TEM	Dry eye disease	Animal model In vitro study	hybrid deliver siRNA	mouse	topical instillation	5 μL	3 times daily	1 week	1 week	1 week
(Yang et al., 2024) [77]	M2 macrophage	DUC	None	TEM, WB, NTA	Dry eye disease	Animal model	treat DED	mouse	topical instillation	5 μL	twice daily	10 days	20 days	20 days
(Yi et al., 2024) [78]	HAEC	DUC, IAC	None	TEM, NTA, SDS-PAGE	Dry eye disease	Animal model In vitro study	treat DED	mouse	topical instillation	5 μL	3 times daily	2 weeks	2 weeks	2 weeks
(Yu et al., 2020) [79]	ADSC	PEG	None	TEM, WB	Dry eye disease	Animal model In vitro study	inhibit NLRP3	mouse	topical instillation	5 μL	4 times daily	5 days	5 days	5 days
(Zhao et al., 2024) [80]	BMSC	DUC	miRNA	TEM, NTA	Dry eye disease	Observational study Animal model Ex vivo study	deliver miR-21-5p	mouse	Intravenous injection (tail vein)	50 μg	every other day	2 weeks	2 weeks	2 weeks
(Zhou et al., 2022) [81]	hUC-MSC, BMSC	DUC	miRNA	TEM, NTA, WB, miRNA-Seq	Dry eye disease	Clinical trial Animal model	miR-204 reprograms M1 to M2	mouse/human	topical instillation	mouse: 5 μL human: 50 μL	mouse: twice daily human: 4 times daily	mouse: 1 week human: 2 weeks	mouse: 1 week human: 2 weeks	mouse: 1 week human: 2 weeks
(Parekh et al., 2021) [82]	HCEnC	DUC	None	NTA, FC, IF	Fuchs’ endothelial corneal dystrophy	In vitro study	inhibit CEC proliferation							
(Parekh et al., 2023) [83]	HCEC	DUC	miRNA	NGS	Fuchs’ endothelial corneal dystrophy	In vitro study	inhibiting miR-195-5p induces HCEC proliferation							
(Ayilam Ramachandran et al., 2023) [84]	Pseudomonas aeruginosa	SEC	Protein	NTA, TEM, MS	Keratitis	In vitro study	disrupt innate immunity							
(Ayilam Ramachandran et al., 2024) [85]	CEC	SEC	None	NTA, WB, TEM, SDS-PAGE	Keratitis	In vitro study	mediate neutrophil chemotaxis							
(Ayilam Ramachandran et al., 2024) [86]	CEC	SEC	Metabolite	NTA, TEM, WB	Keratitis	In vitro study	exploited by PA to deplete PAMC							
(Duan et al., 2024) [87]	Candida albicans	DUC	None	TEM, NTA, SDS-PAGE	Keratitis	Animal model In vitro study	protect against keratitis	mouse	subconjunctival injection	10 μL	single dose	/	5 days	5 days
(Huang et al., 2023) [88]	Tear	DUC	None	SDS-PAGE, WB, DLS, IF	Keratitis	Observational study Ex vivo study	tear exosomes spread HSV-1							
(Lin et al., 2019) [89]	Acanthamoeba castellanii	PEG	Protein	SDS-PAGE, TEM, NTA, LC-MS/MS	Keratitis	In vitro study	induce immune response							
(Ma et al., 2024) [90]	Tear	DUC	Metabolite	NTA, TEM, WB	Keratitis	Observational study Ex vivo study	tear metabolites as HSK indicators							
(Meng et al., 2024) [91]	Aspergillus fumigatus	DUC	None	TEM, NTA, WB, SDS-PAGE	Keratitis	Animal model In vitro study	mitigate fungal keratitis	mouse	subconjunctival injection	10 μL	single dose	/	3 days	3 days
(Yu et al., 2025) [92]	HCEC	DUC	miRNA	TEM, NTA, WB	Keratitis	In vitro study	let-7b-5p promotes M1 activation							
(Hadvina et al., 2023) [93]	HKC, HCF	DUC	miRNA, protein	NTA, TEM, IEM, WB	Keratoconus	Observational study Ex vivo study	altered miRNA/protein profile in KC							
(Hefley et al., 2022) [94]	Tear	None	None	ExoView	Keratoconus	Observational study Ex vivo study	tEVs differ in KC							
(Lozano et al., 2022) [95]	HCK, HKC	PEG	miRNA, protein	NTA, TEM, SDS-PAGE, LC-MS/MS, NGS	Keratoconus	Observational study Ex vivo study	alter stromal cell behavior							
(Ergin et al., 2025) [96]	ADSC	DUC	None	DLS, NTA, TEM	Limbal stem cells deficiency	Animal model	treat LSCD	cat	topical instillation	60 μL/3 mL	4 times daily	15 days	30 days	30 days
(Guo et al., 2022) [97]	Cultured oral mucosal epithelial cell	PEG	miRNA	NTA, TEM, WB	Limbal stem cells deficiency	In vitro study	miRNAs mediate angiogenesis inhibition							
(Li et al., 2025) [98]	ADSC	DUC	miRNA	NTA, SEM, WB, RNA-Seq	Limbal stem cells deficiency	In vitro study	enhance LEC colony formation via miRNAs							
(Ramos et al., 2022) [99]	HCjEC	DUC	miRNA	NTA, FC, NGS	Limbal stem cells deficiency	In vitro study	trigger epithelial transdifferentiation							
(Wang et al., 2023) [100]	CSSC	DUC	miRNA	TEM, NTA, WB, miRNA-Seq	Limbal stem cells deficiency	In vitro study	enhance LESC stemness via Notch							
(Hur et al., 2025) [101]	hUC-MSC	SEC	siRNA	TEM, NTA, WB	Neovascular ocular disease	In vitro study	VEGFA siRNA-loaded induce apoptosis							
(Liu et al., 2025) [102]	Platelet	DUC	Loaded drug	TEM, DLS, NTA	Neovascular ocular disease	Animal model In vitro study	PEV-KM inhibits neovascularization	mouse	topical instillation	5 μL	once daily	1 week	1 week	1 week
(Kistenmacher et al., 2024) [103]	CSSC, LM	TFF, SEC	Protein	NTA, TEM, WB	None	In vitro study	niche cell sEVs regulate limbal stem cell niche							
(Lee et al., 2024) [104]	M1 macrophage	PEG	Protein	NTA	Pterygium	Observational study Ex vivo study	EGF suppresses pterygium inflammation							
(Cortes-Troncoso et al., 2020) [105]	T cell	DUC	miRNA	None	Sjögren’s syndrome	Observational study Ex vivo study	T cell-derived miR-142-3p impairs gland function							
(Kakan et al., 2020) [106]	Serum	DUC	miRNA	TEM, WB, NTA, NGS	Sjögren’s Syndrome	Animal model	serum miRNAs as SS biomarkers							
(Li et al., 2022) [107]	hUC-MSC	DUC	miRNA	NTA, TEM, WB	Sjögren’s Syndrome	Animal model In vitro study	miR-100-5p promotes M2 polarization	rabbit	subconjunctival injection	/	Preventive: days −7, −5, −3 Therapeutic: twice weekly	Preventive: 7 days Therapeutic: 4 weeks	4 weeks	4 weeks
(Liu et al., 2025) [108]	Plasma	SEC, PEG	miRNA	TEM, NTA, WB, miRNA-Seq	Sjögren’s Syndrome	Observational study Ex vivo study	promote Tfh expansion							
(Ma et al., 2023) [109]	hUC-MSC	DUC	None	TEM, NTA, WB	Sjögren’s Syndrome	Observational study Ex vivo study	modulate CD4^+^ T cells							
(Ogata et al., 2024) [110]	iPSC	DUC	miRNA	TEM, WB	Sjögren’s Syndrome	Animal model In vitro study	contain let-7 family miRNAs	mouse	Intravenous injection (tail vein)	300 μg/mL	single dose	/	4 weeks	4 weeks
(Rui et al., 2021) [111]	OE-MSC, BMSC	DUC	Protein	NTA, TEM, SEM, WB, LC-MS/MS	Sjögren’s Syndrome	Animal model In vitro study	enhance MDSC function	mouse	Intravenous injection (tail vein)	100 μg	days 18, 25	/	6 weeks	6 weeks
(Xie et al., 2025) [112]	LG	DGUC	miRNA	TEM, NTA, WB, miRNA-Seq	Sjögren’s Syndrome	Animal model In vitro study	let-7f-5p suppresses Th17 cells	mouse	Intravenous injection (tail vein)	100 μg	three times weekly	2 weeks	8 weeks	8 weeks
(Zhou et al., 2023) [113]	MDSC	DUC, PEG	miRNA	NTA, WB	Sjögren’s Syndrome	Animal model	MDSC-derived deliver miR-10a-5p	mouse	Intravenous injection (tail vein)	100 μg	days 18, 25	/	35 days	35 days
(Zou et al., 2024) [114]	hUC-MSC	DUC	None	EM, WB	Sjögren’s Syndrome	Animal model	regulate gut microbiota and Treg/Th17	mouse	Intravenous injection (tail vein)	/	single dose	/	8 weeks	8 weeks
(Jia et al., 2022) [115]	BMSC	DUC	None	TEM, WB	Transplant rejection	Animal model	prolong graft survival	rat	subconjunctival injection	10 μg/100 μL	days 0, 2	/	until graft rejection (max ~20 days)	/
(Lee et al., 2024) [116]	Serum	SEC	Protein	NTA, WB	Transplant rejection	Animal model	serum protein profiles predict rejection							

* ADSC, Equine Adipose-Derived Stromal Stem Cell; BMSC, bone marrow mesenchymal stem cell; CEC, corneal epithelial cell; CSSC, corneal stromal stem cell; ESC-MSC, embryonic stem cell-derived mesenchymal stem cell; HAEC, human amniotic epithelial cell; HCF, human corneal fibroblasts; HCjEC, conjunctival epithelial cell; HCjGC, human primary conjunctival goblet cell; HCK, human corneal keratocyte; HKC, human keratoconus fibroblast cell; hP-MSC, human placenta-derived MSC; hUC-MSC, human umbilical cord mesenchymal stem cell; iPSC, induced pluripotent stem cell; LESC, limbal epithelial stem cell; LG, labial gland; LM, limbal melanocyte; mAF-MSC, mouse amniotic fluid mesenchymal stem cell; MDSC, myeloid-derived suppressor cell; MSC, mesenchymal stem cell; OE-MSC, olfactory ecto-mesenchymal stem cell; PDLSC, periodontal ligament mesenchymal stem cell; # DUC, Differential sequential ultracentrifugation; DGUC, Density gradient ultracentrifugation; PEG, Polyethylene glycol precipitation; TFF, Tangential flow filtration; SEC, Size exclusion chromatography; IAC, Immunological affinity capture; ^ WB, Western blot; MS, Mass spectrometry; LC-MS/MS, Liquid Chromatography–Tandem Mass Spectrometry; ICP-MS, Inductively coupled plasma mass spectrometry; IF, Immunofluorescence microscopy; ELISA, Enzyme-linked immuno-sorbent assay; EM, Electron microscopy; TEM, Transmission EM; IEM, Immuno-EM; SEM, Scanning EM; STED, Stimulated emission depletion nanoscopy; EDS, Energy dispersive spectroscopy; FC, Flow cytometry; NTA, Nanoparticle tracking analysis; DLS, Dynamic light scattering; TRPS, Tunable resistive pulse sensing analysis; miRNA-Seq, microRNA sequencing; AFM, Atomic force microscopy; SDS-PAGE, SDS protein gel electrophoresis; RNA-Seq, RNA sequencing; NGS, Next-generation sequencing.

## Data Availability

No new data were created or analyzed in this study.

## Abbreviations

The following abbreviations are used in this manuscript:

AK	acanthamoeba keratitis
AC	allergic conjunctivitis
APC	antigen-presenting cell
ADDE	aqueous-deficient dry eye
AF	aspergillus fumigatus
BC	bacillus coagulans
BAC	benzalkonium chloride
BMSC	bone marrow-derived MSC
CGCs	conjunctival goblet cells
CSSCs	corneal stromal stem cells
COMECs	cultured oral mucosal epithelial cells
DAMPs	damage-associated molecular patterns
DCs	dendritic cells
DGUC	density Gradient Ultracentrifugation
DUC	differential Ultracentrifugation
DEGMA	dimethylethylene glycol dimethacrylate
DHA	docosahexaenoic acid
DED	dry eye disease
ER	endoplasmic reticulum
ESCRT	endosomal sorting complex required for transport
EGF	epidermal growth factor
EMT	epithelial–mesenchymal transition
EDE	evaporative dry eye
ECM	extracellular matrix
EVs	extracellular vesicles
Fbxw7	F-box and WD repeat domain-containing 7
FAM	fluorescein amide
FECD	Fuchs’ endothelial corneal dystrophy
FK	fungal keratitis
FM	fungal metabolite
GelMA	gelatin methacrylate
HSK	herpes simplex keratitis
HSV-1	herpes simplex virus type 1
hADSC	human adipose-derived stem cell
HCEnCs	human corneal endothelial cells
HCECs	human corneal epithelial cells
HCFs	human corneal fibroblasts
HCKs	human corneal keratocytes
HCMs	human corneal myofibroblasts
hUCMSC	human umbilical cord mesenchymal stem cell
HA	hyaluronic acid
IAC	immunological Affinity Capture
iPSC	induced pluripotent stem cell
ILVs	intraluminal vesicles
KC	keratoconus
LESCs	limbal epithelial stem cells
LMs	limbal melanocytes
LNCs	limbal niche cells
LSCD	limbal stem cell deficiency
MMPs	matrix metalloproteinases
MSCs	mesenchymal stem cells
MISEV2023	Minimal Information for Studies of Extracellular Vesicles
MAPK	mitogen-activated protein kinase
MWCO	molecular weight cut-offs
mADSC	mouse adipose-derived stem cell
mAF-MSC	mouse amniotic fluid-derived mesenchymal stem cell
MVBs	multivesicular bodies
MDSCs	myeloid-derived suppressor cells
NOD	non-obese diabetic
NF-κB	nuclear factor kappa B
OSDs	ocular surface diseases
OE	olfactory ecto
PDLSC	periodontal ligament stem cell
PEG	polyethylene glycol
PA	pseudomonas aeruginosa
ROS	reactive oxygen species
Treg	regulatory T cells
SEC	size Exclusion Chromatography
SS	Sjögren’s syndrome
sEVs	small extracellular vesicles
SPMs	specialized pro-resolving mediators
Tfh	T follicular helper cells
Th17	T helper 17 cells
THH	thermosensitive hydrogel
TSP-1	thrombospondin-1
UPR	unfolded protein response
VEGFR1	vascular endothelial growth factor receptor 1
